# Investigating the impact of the COVID‐19 pandemic on older adolescents' psychological wellbeing and self‐identified cognitive difficulties

**DOI:** 10.1002/jcv2.12164

**Published:** 2023-05-29

**Authors:** Meg Attwood, Christopher Jarrold

**Affiliations:** ^1^ School of Psychological Science University of Bristol Bristol UK

**Keywords:** adolescence, anxiety, cognitive function, pandemic, wellbeing

## Abstract

**Background:**

The COVID‐19 pandemic coincides with growing concern regarding the mental health of young people. Older adolescents have faced a particular set of pandemic‐related challenges and demonstrate heightened vulnerability to affective disorders (particularly anxiety). Anxiety symptoms are associated with a range of cognitive difficulties. Older adolescents may therefore be susceptible to pandemic‐related declines in wellbeing and associated cognitive difficulties.

**Methods:**

At three timepoints, independent samples of young people aged 16–18 years (*N* = 607, 242, 618 respectively) completed an online survey. Data collection coincided with periods of lockdown (timepoints 1 and 3) and young people returning to school (timepoint 2). The survey assessed subjective impacts of the pandemic on overall wellbeing, anxiety and cognitive function.

**Results:**

Findings demonstrated the detrimental impact of the COVID‐19 pandemic on older adolescents' psychological wellbeing—a finding that was consistent across samples. The majority of young people at each timepoint experienced heightened anxiety. Crucially, pandemic‐related anxiety was associated with self‐identified cognitive difficulties, a pattern of association that was evident at all three timepoints. The nature and extent of these difficulties were predictive of specific pandemic‐related concerns in this age group.

**Conclusions:**

Older adolescents' experiences of the pandemic are characterised by subjective declines in wellbeing and stable patterns of association between anxiety and self‐identified cognitive difficulties. Implications are discussed with reference to future research and intervention.

## Graphical abstract

Detrimental impacts of the COVID‐19 pandemic on older adolescents' wellbeing were evident during periods of lockdown and when restrictions eased. A pattern of association was identified between pandemic‐related anxiety and self‐identified cognitive difficulties. The nature and extent of these difficulties predicted the types of worries reported by young people.

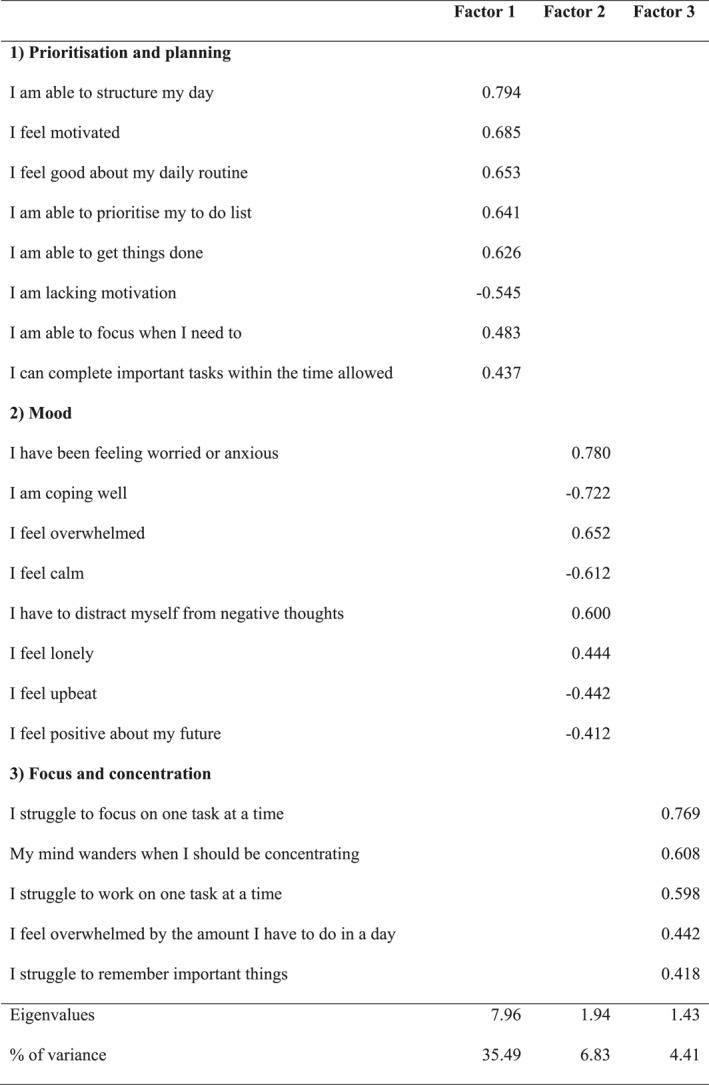

**Key points**

- There is growing concern regarding the detrimental impact of the COVID‐19 pandemic on young people's mental health and wellbeing.
- Older adolescents are vulnerable to the onset of emotional difficulties and have faced a particular set of pandemic‐related challenges.
- This study demonstrates the detrimental impact of the pandemic on older adolescents' psychological wellbeing and crucially, supports a clear and stable pattern of association between anxiety and self‐identified cognitive difficulties in this age group (a novel finding).
- These findings have implications for both our theoretical understanding of adolescent anxiety and assessment and intervention approaches for vulnerable young people.

## INTRODUCTION

The COVID‐19 pandemic has resulted in significant, sustained disruption to the education, social interaction and daily routines of young people across the globe. While the SARS‐CoV‐2 virus poses limited risk to adolescents' physical health (Bhopal et al., 2020), there is considerable concern regarding the impact of the pandemic on young people's psychological wellbeing (Crawley et al., 2020; Orben et al., 2020). Mental health difficulties typically emerge in adolescence (Merikangas et al., 2010) and psychosocial experiences are fundamental in shaping young people's risk (and resilience) to these difficulties (Paus et al., 2008; Rapee et al., 2019). Social distancing measures introduced significant “social risk factors” for poor mental health in young people (Dewa et al., 2021, p. 667).

There is growing recognition of the vulnerability of older adolescents to mental health difficulties. For example, in the United Kingdom (UK), rising rates of prevalence of emotional difficulties (most commonly, anxiety) have been reported in young people (particularly girls) aged 17–19 (Sadler et al., 2018). Given their proximity to school leaving; school closures, examination cancellations, and uncertainty regarding jobs and university places may have been particularly anxiety‐provoking for this age group. Indeed, disproportionate negative effects of the pandemic on the wellbeing of older adolescents/young adults, including girls and individuals with pre‐existing mental health difficulties (e.g., Fancourt et al., 2021; Racine et al., 2021; Smirni et al., 2020) were reported early on in the pandemic.

Rising rates of prevalence of adolescent affective disorders (e.g., Collishaw, 2015) pre‐date the pandemic, with anxiety the most common of these difficulties (Polanczyk et al., 2015). While anxiety and worry are typical (and adaptive) responses to environmental stressors (Eysenck & Calvo, 1992), persistent elevated levels of anxiety are associated with significant impairment in functioning across psychosocial domains (Baxter et al., 2014). In the cognitive domain, anxiety‐related difficulties are well‐established (Moran, 2016) and include difficulties with concentration (Airaksinen et al., 2005), impairments in reading comprehension (Calvo et al., 1992) and problem‐solving ability (Ashcraft & Kirk, 2001), and lower performance on standardised assessments of intelligence (Eysenck et al., 2007). Cognitive difficulties may underpin the increased likelihood of academic underachievement in anxious young people (Van Ameringen et al., 2003).

The pandemic has been marked by prolonged educational disruption and sustained uncertainty regarding both its course and the measures required to contain it. Investigating the extent to which these challenges are associated with detrimental impacts on adolescents' cognitive function will inform our understanding of anxiety‐related cognitive difficulties in this age group and targeted intervention for vulnerable young people. While several studies have sought to understand the impact of COVID‐19 infection on individuals' cognitive functioning (e.g., Becker et al., 2021; Daroische et al., 2021), to our knowledge only one study has investigated the implications of social distancing measures for cognitive functioning (Ingram et al., 2021). This provides initial support for an association between social distancing measures (enforced social isolation during a period of lockdown) and reduced cognitive performance in an adult sample (aged 18–72 years). Although prior research has typically focused on older adults (Evans et al., 2019), studies with younger participants have demonstrated that even perceived social exclusion can impair cognitive function (Baumeister et al., 2005) and self‐regulation (Twenge et al., 2001). While there is initial support for an association between social isolation and cognitive impairment during the pandemic (Ingram et al., 2021), this has yet to be examined in an adolescent cohort or during periods of less extreme social distancing measures.

The current study investigated the impact of the COVID‐19 pandemic on older adolescents' psychological wellbeing and self‐identified cognitive difficulties during periods of school closure during lockdown and on returning to school. The aims were: to determine the subjective impact of the pandemic on older adolescent wellbeing and, crucially, to assess the extent to which any detrimental wellbeing impacts were associated with self‐identified cognitive difficulties.

## METHODS

### Participants

Participants aged 16–18 years were eligible to take part in this study. A database of UK^1^ secondary schools and sixth form colleges was created using local authority school finder websites and young people were recruited via email contact with over 400 schools/colleges. Data were collected at three timepoints from independent samples of young people and recruitment methods were consistent at each timepoint. The first survey was conducted in June and July 2020 when UK secondary schools and colleges were closed as part of national lockdown measures (timepoint 1). The second survey took place in September 2020 when students returned to school after the summer break (timepoint 2); and the third survey was conducted in February and March 2021 during another period of school closures (timepoint 3). A detailed description of relevant social distancing measures in place during this 12‐month period is provided in Appendix S1.1.

### Measures

The study utilised a cross‐sectional survey design in response to the rapid onset and changing circumstances of the pandemic. The survey was divided into subsets of items, which assessed overall wellbeing, anxiety and worries, cognitive function, and school experience (see Appendix S1.2 for the full survey schedule). Participants provided self‐report assessments of their functioning using a combination of visual analogue scales (VAS), Likert‐scale items and standardised measures of trait and pandemic‐related anxiety.^2^ VAS items, which are routinely included in psychological studies to capture global or “state” measures of psychological constructs (e.g., pain, anxiety, wellbeing), provided single‐item measures of overall wellbeing and anxiety. Likert‐scale items, which included both positive and negatively‐worded statements, were rated on a 4‐point scale (“never”, “some of the time”, “most of the time”, “always”), unless otherwise specified. Demographic data including age, gender, year group (Year 11, 12 or 13 corresponding to the final three years of formal schooling), country of domicile and current school attendance were captured. Survey items (except where specified) and item order remained consistent across timepoints.^3^ Subtle changes to the wording of some items were made to account for the changing context (i.e., learning from home or returning to school).

### Wellbeing

The first survey subsection focused on young people's overall wellbeing. Participants were asked to provide a global, single‐item rating of their wellbeing on a VAS from 0 to 10 and to report any pandemic‐related changes in wellbeing (increase, decrease or no change). Participants reporting decreased wellbeing identified the main contributor to this change by selecting from a prespecified list (or adding their own contributors). Participants also provided Likert‐scale ratings for different aspects of their mood (e.g., “I feel lonely”, “I feel positive about my future”) and reported on the quality of their sleep, social interaction and physical activity (providing a rating in each case of “too much”, “too little” or “the right amount”).

### Anxiety and worries

The second subsection focused on participants' anxiety and worries. Participants rated their level of anxiety (over the past week) on a VAS from 0 to 10, and reported any pandemic‐related changes in anxiety (increase, decrease or no change). Participants reporting pandemic‐related anxiety then selected and ranked items from a prespecified list (adding their own items where applicable) to identify contributors to anxiety and associated impacts (see item A5 in Appendix S1.2). All participants were then presented with a list of possible concerns (e.g., concerns regarding the future, job prospects, schoolwork) and asked to select those that were applicable to them.^4^

Participants also completed the trait scale of the State‐Trait Inventory of Cognitive and Somatic Anxiety (STICSA; Ree et al., 2000), which assesses trait vulnerability to anxiety. This 21‐item measure produces a total trait anxiety score and subscale scores for cognitive and somatic dimensions of trait anxiety. The scale demonstrated good reliability in all three samples (*α* = 0.92; α = 0.92; α = 0.91 respectively). Surveys 2 and 3 included the 7‐item Pandemic Anxiety Scale (McElroy et al., 2020), a newly developed measure that assesses anxieties relating to COVID‐19 infection (e.g., “I am worried that I will catch COVID‐19”) and consequences of the pandemic (e.g., “I am worried about missing school”). The scale demonstrated acceptable reliability in both samples (*α* = 0.69; α = 0.67).

### Cognitive function

In the penultimate section, participants rated their level of functioning in relation to two cognitive domains: attention (e.g., “My mind wanders when I should be concentrating”), and planning/task completion (e.g., “I am able to prioritise my to do list”). These core cognitive skills were chosen because of their particular relevance to young people's ability to cope during periods of prolonged school closures and independent learning. No reference was made in this section to anxiety or wellbeing.

### School engagement

The final survey items focused on school experience and captured general feelings about schoolwork, and subjective impacts of the pandemic on learning. Participants identified any difficulties with distance learning or access to online materials. At timepoint 1, participants also reported on the regularity of teacher contact.

### Ethical considerations

Participating settings shared a study flyer with eligible students, which included a weblink with detailed study information. Informed consent was obtained from all participants prior to survey completion. Full ethical approval for the study was provided by the School of Psychological Science's Human Research Ethics Committee at the University of Bristol (ref: 104404). Participants who consented were entered into a prize draw for an e‐voucher of £100.

### Data analysis

Initial descriptive analyses characterised subjective impacts of the pandemic on respondents' wellbeing. Subsequent correlational analyses assessed relationships between wellbeing and anxiety (state, trait), and independent *t*‐tests identified gender‐related wellbeing differences within each sample. Pandemic‐related impacts of anxiety (where present) were examined by visualising proportional ranked responses in a heatmap. Identified associations between anxiety and domains of functioning at timepoint 1 were further elucidated using exploratory factor analysis (EFA). Given the collection of two subsequent samples of data (at timepoints 2 and 3), this EFA factor structure could be validated using confirmatory factor analysis (CFA). Subsequent regression analyses (using regression scores from the factor analyses) added specificity to the relationship between anxiety and self‐identified difficulties by relating these to particular pandemic‐related concerns (e.g., worries about schoolwork) at each timepoint.

## RESULTS

Final samples for each study were as follows: 607 participants (392 girls, *M* = 17.55 years) at timepoint 1; 242 participants (172 girls, *M* = 17.15 years) at timepoint 2; and 618 participants (465 girls, *M* = 17.24 years) at timepoint 3. Sample characteristics and all preliminary screening measures are reported in full (see Appendix S1.3). For brevity, given the scope of the study, results relating to the main study aims are reported below, and contextual information and supplementary analyses are included in the online Supporting Information.

### Preliminary analyses

Mean wellbeing and anxiety scores for girls and boys are presented in Table 1. Preliminary analyses at timepoint 1 identified significant positive correlations between state (VAS) and trait (STICSA) anxiety measures (*r* = 0.634, *p* < 0.001) and negative correlations between wellbeing (VAS) and state and trait anxiety measures (*r* = −0.549, *p* < 0.001; *r* = −0.550, *p* < 0.001). These relationships were consistent across samples (Appendix S1.4). Gender differences in wellbeing were demonstrated at each timepoint, with girls consistently reporting lower wellbeing and higher anxiety scores than boys (Appendix S1.5).

**TABLE 1:** Wellbeing and anxiety scores by gender at successive timepoints.

	Study 1		Study 2		Study 3	
	M	SD	M	SD	M	SD
Trait anxiety (STICSA total)*						
Female	45	11.80	48.24	11.53	48.48	11.16
Male	38.20	10.01	36.37	10.42	39.80	11.09
State anxiety (VAS)						
Female	5.43	2.33	6.10	2.34	5.91	2.15
Male	4.17	2.30	4.55	2.85	5.15	2.40
Overall wellbeing (VAS)						
Female	5.32	1.85	5.37	1.94	4.75	1.92
Male	6.18	1.84	6.08	1.95	5.58	1.94

*Note*: A score of 43 on this trait scale denotes “probable cases of clinical anxiety” and a threshold score of 40 is applied for “possible cases of clinical anxiety” (Roberts et al., 2016).

### Impacts of the pandemic on older adolescent wellbeing and anxiety

When asked to report the impact of the pandemic on their wellbeing, the majority of young people at each timepoint reported decreased wellbeing (62%, 58%, 76% respectively). Girls were more likely to report subjective declines in wellbeing than boys, a finding that was consistent across samples (Appendix S2.1). Reduced social contact, anxiety/worries, and uncertainty about the future were cited as predominant contributors to this decline and reports of low mood, negativity about the future, loneliness and difficulty coping were common at each timepoint. Negative impacts of the pandemic on sleep, physical activity, and social interaction were also reported (Appendix S2.2).

At each timepoint, the majority of respondents reported pandemic‐related increases in anxiety (58%, 63%, 71% respectively). Supplementary analyses (Appendix S3.1) demonstrate that state anxiety scores were consistently higher for individuals reporting increased anxiety on account of the pandemic. Participants were asked to select and rank contributors to their anxiety (where present) and proportional ranked responses were examined to identify predominant contributors (Appendix S3.2). Uncertainty about the future was the main contributor to anxiety at all three timepoints followed by limited social contact during periods of lockdown, and examination disruption and school concerns as young people returned to school (concerns which were consistent at timepoint 3).

Participants identified the nature of their concerns (e.g., worries about schoolwork, their own or others wellbeing, or job prospects) at each timepoint (Appendix S3.3). The most commonly reported worry at all timepoints (for boys and girls) was concern about the future. Girls' most common concerns related to uncertainty about the future, schoolwork and their own psychological wellbeing. During periods of lockdown (timepoints 1 and 3), concerns regarding schoolwork were common among boys, but on returning to school (timepoint 2), worries about the health of others and job prospects became more prominent.

### Examining associations between anxiety and cognitive difficulties

Participants were asked to identify areas of their functioning most affected by anxiety. Proportional ranked responses were examined to identify predominant impacts (Appendix S4.1). At timepoints 1 and 2, respondents most commonly reported effects of anxiety on their mood, motivation, and focus and concentration. At timepoint 3, overall wellbeing was most commonly cited, followed by motivation, mood, and focus and concentration.

In order to elucidate these reported associations between anxiety and self‐identified cognitive difficulties (relating to motivation and focus and concentration), survey items from timepoint 1 (including items from the wellbeing, anxiety, and cognition subsets^5^) were entered into an EFA. Any respondents who omitted more than 2 of the 22 survey items included in the EFA were dropped from subsequent analyses, which reduced the sample to 606 respondents.^6^ The use of EFA in this instance was supported by a KMO value above 0.9 and highly significant Bartlett's test (*p* < 0.001). Visual inspection of the scree plot (following an initial EFA without rotation) suggested a three factor solution, which was confirmed with a subsequent parallel analysis (Patil et al., 2017). The final EFA, estimated using Principal Axis Factoring and Direct Oblimin rotation, produced three factors (with eigenvalues >1), which explained a total of 47% of the variance in survey responses. 21 of the 22 items had sufficiently high loadings (>0.4) to be included in the model (see Table 2). Item loadings supported characterisation of factors 1 and 3 as cognitive factors describing skills relating to prioritisation and planning, and focus and concentration respectively, while factor 2 describes participant mood. Factor scores relating to each area of self‐identified difficulty (factors 1, 2 and 3) were computed for each respondent using the regression method.

**TABLE 2:** Factor loadings after rotation (exploratory factor analysis (EFA), timepoint 1).

	Factor 1	Factor 2	Factor 3
1) Prioritisation and planning			
I am able to structure my day	0.794		
I feel motivated	0.685		
I feel good about my daily routine	0.653		
I am able to prioritise my to do list	0.641		
I am able to get things done	0.626		
I am lacking motivation	−0.545		
I am able to focus when I need to	0.483		
I can complete important tasks within the time allowed	0.437		
2) Mood			
I have been feeling worried or anxious		0.780	
I am coping well		−0.722	
I feel overwhelmed		0.652	
I feel calm		−0.612	
I have to distract myself from negative thoughts		0.600	
I feel lonely		0.444	
I feel upbeat		−0.442	
I feel positive about my future		−0.412	
3) Focus and concentration			
I struggle to focus on one task at a time			0.769
My mind wanders when I should be concentrating			0.608
I struggle to work on one task at a time			0.598
I feel overwhelmed by the amount I have to do in a day			0.442
I struggle to remember important things			0.418
Eigenvalues	7.96	1.94	1.43
% of variance	35.49	6.83	4.41

Though distinguishable, these three factors were significantly correlated such that increased difficulties with mood (factor 2) were associated with reduced ability to plan and prioritise (factor 1; *r =* −0.572, *n* = 606, *p* < 0.001) and greater difficulty with focus and concentration (factor 3; *r* = 0.520, *n* = 606, *p* < 0.001). A multiple regression analysis established the association between self‐identified mood and cognitive difficulties, and trait anxiety scores. Together these factors explained 56% of the variance in trait anxiety scores (*F* (3, 602) = 262.171, *p* < 0.001, *r* ^2^ = 0.564). Factors 2 and 3 contributed statistically significantly to the model, *p* < 0.001, while Factor 1 did not (*p* = 0.503).

**FIGURE 1:**
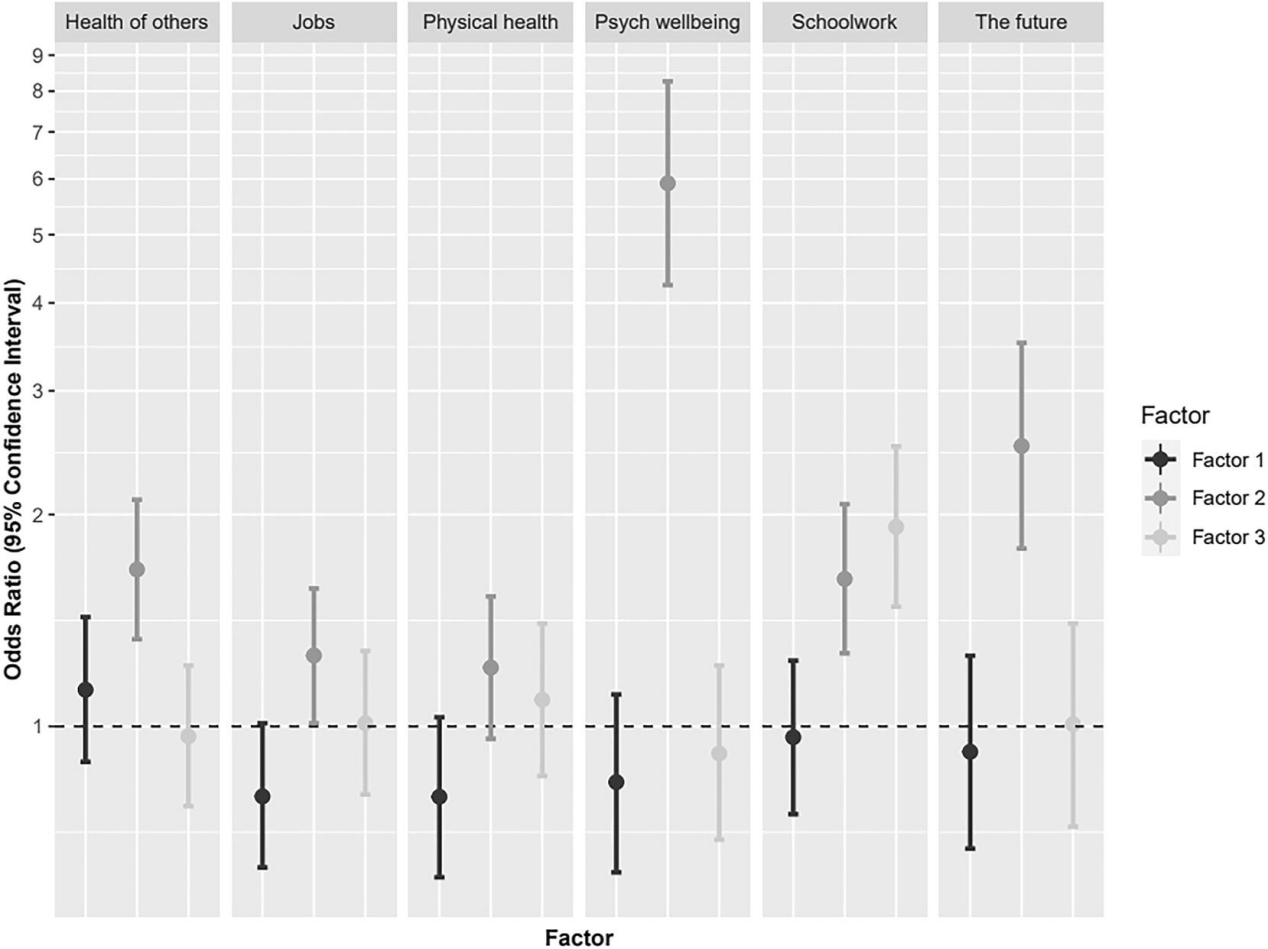
Logistic regression results demonstrating likelihood of worries as predicted by exploratory factor analysis (EFA) factor scores (timepoint 1).

In order to add specificity to this pattern of association between anxiety and self‐identified difficulties, a subsequent series of logistic regression analyses examined the extent to which self‐identified difficulties (as indexed by regression scores from the EFA) were related to specific concerns at timepoint 1 (see Figure 1, and Appendix S4.2 for full model statistics, as well as Appendix S4.4 for results of a complementary set of ordinal regression analyses at timepoint 3). While mood difficulties were predictive of all but one of the reported worries, the strength of their predictive value varied across concerns. Mood difficulties were most predictive of concerns about respondents' own psychological wellbeing and the future. Difficulties with focus and concentration and of mood were predictive of worries about schoolwork, although the former was the stronger predictor. Planning and prioritisation was not a significant predictor of any concerns at this timepoint.

### Cross‐validating associations between anxiety and self‐identified cognitive difficulties

**FIGURE 2:**
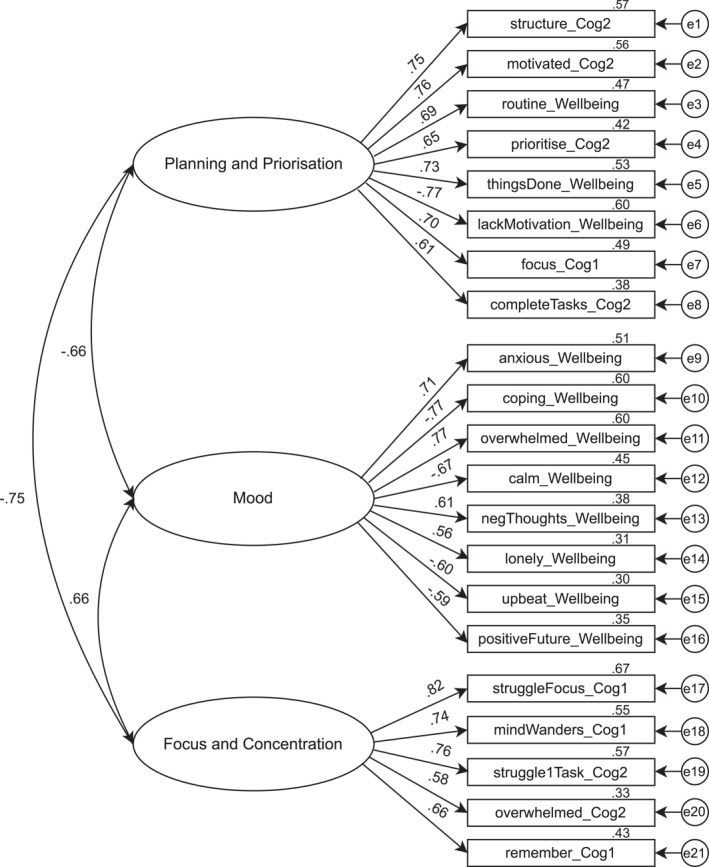
Confirmatory factor analysis path diagram (timepoint 3).

Subsequent confirmatory factor analyses (CFAs) sought to clarify the extent to which the initial EFA factor structure, derived from participants assessed at timepoint 1, was representative of the experience of participants assessed at timepoints 2 or 3. The normality of item distributions was assessed in each case, and no issues were identified with regard to either skew or kurtosis (Kline, 2005). CFAs using maximum likelihood estimation were computed in AMOS (Version 27.0; Arbuckle, 2021) using the EFA factor structure and survey data from timepoints 2 and 3. Any respondents who omitted more than 2 of the 21 survey items included in this analysis were excluded.^7^ Multivariate outliers^8^ were also removed prior to analysis and this produced a final sample of 234 respondents for timepoint 2 and 609 participants for timepoint 3. Table 3 reports the model fit indices from these analyses. Data from timepoints 2 and 3 cross‐validated the 3‐factor structure identified in the EFA, with favourable fit (Brown, 2006; Kline, 2005), and strong factor loadings (Figure 2), which were significant (*p* < 0.001) for all indicator variables.

**TABLE 3:** Model Fit Indices (confirmatory factor analysis (CFA), timepoints 2 and 3).

CFA model	X^2^ (*df)*	*p*	CMIN/DF	TLI	CFI	RMSEA (90% CI)	SRMR
Timepoint 2 (*N* = 234)							
3‐Factor model	397.93 (185)	**	2.15	0.90	0.91	0.070 [0.061, 0.080]	0.0567
Timepoint 3 (*N* = 609)							
3‐Factor model	692.00 (186)	**	3.72	0.91	0.92	0.067 [0.062, 0.072]	0.0569

Abbreviations: CFI, comparative fit index; CMIN/DF, chi‐square fit statistics/degrees of freedom; RMSEA, root mean square of approximation; SRMR, standardized root mean square residual; TLI, Tucker‐Lewis index.

Regression scores were computed for each factor to enable further analysis. A follow‐up multiple regression analysis demonstrated that at timepoint 3, the three (CFA) factors explained 59% of the variance in trait anxiety (*F* (3, 605) = 284.204, *p* < 0.001, *r* ^2^ = 0.585) and all three factors added statistically significantly to the model, *p* < 0.001. At timepoint 2, the three‐factor structure resulted in significant multicollinearity between factors 1 and 3 (the cognitive factors), an issue which affected the accuracy of subsequent regression analyses for timepoint 2.^9^

## DISCUSSION

In addition to demonstrating stable, detrimental impacts of the pandemic on older adolescents' overall wellbeing, the current findings support a clear, cross‐validated pattern of association between anxiety and self‐identified cognitive difficulties during the pandemic. In relation to the first of these findings, the majority of older adolescents reported pandemic‐related declines in their overall wellbeing at all three timepoints, suggesting that detrimental effects of the pandemic were not confined to periods of lockdown. In line with previous work demonstrating disproportionate negative effects of the pandemic on the mental health of older adolescents and young adults (e.g., Fancourt et al., 2021; Smirni et al., 2020), at each timepoint, the majority of respondents reported pandemic‐related increases in anxiety. Young people consistently attributed their anxiety to feelings of uncertainty about the future with other key contributors (e.g., limited social interaction, disrupted routines, worries about returning to school) likely reflecting shifts in the nature of restrictions. Rising rates of prevalence of emotional difficulties in older adolescents pre‐date the pandemic (Sadler et al., 2018). However, incidence of affective symptoms in this age group has continued to increase over its course (NHS Digital, 2020), as has demand for specialist intervention (NHS Digital, 2021). Again, in line with previous findings (e.g., Racine et al., 2021), our data suggest a disproportionate effect of the pandemic on the wellbeing of girls. Girls were more likely to report pandemic‐related increases in anxiety and had consistently higher state anxiety scores and lower overall wellbeing scores than boys. Although these findings likely reflect a pre‐existing bias in girls' susceptibility to emotional difficulties (Sadler et al., 2018), girls' reports of detrimental effects on their wellbeing were consistent across study measures and stable across samples. Anxiety symptoms that emerge in adolescence tend to be more severe and enduring (Essau et al., 2014). As such, it is important that the extent of the challenges faced by this age group are recognised, and targeted intervention put in place to identify and support those most affected.

A crucial finding relates to the identification of a consistent pattern of association between anxiety and self‐identified cognitive difficulties (relating to difficulties with focus and concentration and reduced ability to plan and prioritise tasks) in this population. This pattern of association emerged early on in the pandemic (timepoint 1) and was stable at successive timepoints (even as young people returned to school and pressures associated with distance learning eased). This suggests that self‐identified cognitive difficulties in this age group reflect a sustained impact of the pandemic, as opposed to being linked to particular social distancing protocols (e.g., lockdowns; Ingram et al., 2021). However, a key question, that cannot be answered with these data, is the extent to which subjective reports of cognitive difficulties were accompanied by objective declines in cognitive performance (Soto et al., 2020). Given established links between anxiety, cognitive difficulties and academic underachievement and related work linking enforced social isolation with cognitive impairment in adults (Ingram et al., 2021), future studies should examine the extent to which pandemic‐related cognitive difficulties are evident beyond self‐report, and sustained as the pandemic eases. The current findings identify a role for cognitive difficulties in shaping young people's experiences of the pandemic (both as a subjective consequence of increased anxiety, and as a predictor of worries), and suggest that along with gender‐specific vulnerability, cognitive difficulties may influence individual risk (or resilience) to stress in this age group. Future research could look to examine these risk factors in relation to clinical vulnerability (e.g., anxiety disorder diagnoses): integrating clinical and population‐level data to assess the extent to which these difficulties are characterised by qualitatively different patterns of symptom expression or fall on a continuum.

These findings are limited in certain respects. While our samples are not necessarily representative, care was taken to approach schools and colleges across the UK in order to capture the variety of experiences of older adolescents. However, we did not collect sociodemographic data as part of our survey and therefore cannot draw any conclusions regarding particular effects of the pandemic for those in different socioeconomic groups. While we note the smaller sample size at timepoint 2, this sample was still adequately powered to detect effects of interest, and though sample characteristics did differ, the practical significance of these small differences is questionable (see Appendix S5). Our samples are characterized by high levels of trait anxiety (Roberts et al., 2016), which could suggest recruitment bias, but likely reflects the acknowledged vulnerability of older adolescents (particularly girls) to emotional difficulties and our predominantly female samples. The lack of baseline data also prevents us from establishing whether reports of increased anxiety reflect a “true increase” from pre‐pandemic levels. However, our findings align with those from longitudinal studies that demonstrate increased prevalence of anxiety in young people on account of the pandemic (Kwong et al., 2021; Pierce et al., 2020).

Despite these limitations, this study is novel in its particular focus on cognitive difficulties as experienced by older adolescents during the pandemic. While the cross‐sectional nature of this study limits conclusions that can be drawn regarding shifts in wellbeing and anxiety over the course of the pandemic, capturing survey data from independent samples of young people has enabled a cross‐validation of the identified pattern of association between elevated anxiety and self‐identified cognitive difficulties (first identified at timepoint 1). Our findings therefore demonstrate clear, stable patterns of association between heightened anxiety and self‐identified cognitive difficulties in this age group during the pandemic.

## CONCLUSION

Older adolescents have acknowledged vulnerability to emotional difficulties and have faced a particular set of pandemic‐related challenges. The findings of this study demonstrate detrimental impacts of the pandemic on the wellbeing of older adolescents that were present during periods of lockdown and on returning to school and support a clear pattern of association between anxiety and self‐identified cognitive difficulties in this age group. Further work is required to identify how to enable young people at a crucial stage in their education (and broader development) to cope with sustained uncertainty ‐ uncertainty that reflects significant societal challenges, not limited to COVID‐19. While this study focused on the context of a pandemic, the findings can therefore inform ongoing work determining the broader impacts of stress, both acute (e.g., during examination periods) and chronic (e.g., climate anxiety), on young people's psychological functioning, and to shape assessment and intervention for those most vulnerable to anxiety.

## AUTHOR CONTRIBUTIONS

**Meg Attwood**: Conceptualization; Data curation; Formal analysis; Funding acquisition; Methodology; Project administration; Writing—original draft. **Christopher Jarrold**: Conceptualization; Funding acquisition; Methodology; Supervision; Writing—review & editing.

## CONFLICT OF INTEREST STATEMENT

The authors have declared that they have no competing or potential conflicts of interest.

## Supporting information

Supporting Information S1

## Data Availability

Supplementary analyses and contextual information in support of this submission are provided in the online supplement. Due to the sensitivity of the data involved, these data are published as a controlled dataset at the University of Bristol Research Data Repository data.bris, at https://doi.org/10.5523/bris.2nit8ium1cuwy2merjyyoe2r47. The metadata record published openly by the repository at this location clearly states how data can be accessed by bona fide researchers. Requests for access will be considered by the University of Bristol Data Access Committee, who will assess the motives of potential data re‐users before deciding to grant access to the data. No authentic request for access will be refused and re‐users will not be charged for any part of this process.

## ENDNOTES

^1^
Schools and colleges in England, Wales and Scotland were approached, but the majority of participants were from England.

^2^
The Pandemic Anxiety Scale (PAS; McElroy et al., 2020) was included at timepoints 2 and 3.

^3^
In an individual differences study, order counterbalancing introduces additional variance and can confound attempts to distinguish between individuals on variables of interest.

^4^
At timepoint 1, participants responded yes or no to identify the presence or absence of these concerns. At timepoints 2 and 3, participants reported their level of concern using a 4‐point Likert scale (never, some of the time, most of the time, always).

^5^
Schoolwork‐related items were excluded because of high levels of missing data (Year 13 respondents had no schoolwork set at timepoint 1). An initial factor analysis with no rotation identified three items (relating to sleep, socialising and exercise) with multiple low‐order correlations (*r* < +/− 0.30). These items were excluded from the EFA (Yong & Pearce, 2013).

^6^
Remaining missing values were imputed using mean substitution for that subscale.

^7^
Remaining missing values (of which there were few) were imputed using mean substitution for that subscale.

^8^
Mahalanobis Distance scores (computed for the 21 survey items included in the CFA) were used to identify multivariate outliers, and these cases were removed from subsequent analyses.

^9^
Appendix S4.3 includes model fit indices for a 2‐factor solution for timepoint 2, which reduced multicollinearity issues, along with subsequent regression analyses.

## References

[jcv212164-bib-0001] Airaksinen, E. , Larsson, M. , & Forsell, Y. (2005). Neuropsychological functions in anxiety disorders in population‐based samples: Evidence of episodic memory dysfunction. Journal of Psychiatric Research, 39(2), 207–214. 10.1016/j.jpsychires.2004.06.001 15589570

[jcv212164-bib-0002] Arbuckle, J. L. (2021). Amos (version 27.0) [computer program]. IBM SPSS.

[jcv212164-bib-0003] Ashcraft, M. H. , & Kirk, E. P. (2001). The relationships among working memory, math anxiety, and performance. Journal of Experimental Psychology: General, 130(2), 224–237. 10.1037/0096-3445.130.2.224 11409101

[jcv212164-bib-0004] Baumeister, R. F. , DeWall, C. N. , Ciarocco, N. J. , & Twenge, J. M. (2005). Social exclusion impairs self‐regulation. Journal of Personality and Social Psychology, 88(4), 589–604. 10.1037/0022-3514.88.4.589 15796662

[jcv212164-bib-0005] Baxter, A. J. , Vos, T. , Scott, K. M. , Ferrari, A. J. , & Whiteford, H. A. (2014). The global burden of anxiety disorders in 2010. Psychological Medicine, 44(11), 2363–2374. 10.1017/s0033291713003243 24451993

[jcv212164-bib-0006] Becker, J. H. , Lin, J. J. , Doernberg, M. , Stone, K. , Navis, A. , Festa, J. R. , & Wisnivesky, J. P. (2021). Assessment of cognitive function in patients after COVID‐19 infection. JAMA Network Open, 4(10), e2130645. 10.1001/jamanetworkopen.2021.30645 34677597 PMC8536953

[jcv212164-bib-0007] Bhopal, S. , Bagaria, J. , & Bhopal, R. (2020). Children's mortality from COVID‐19 compared with all‐deaths and other relevant causes of death: Epidemiological information for decision‐making by parents, teachers, clinicians and policymakers. Public Health, 185, 19–20. 10.1016/j.puhe.2020.05.047 32516623 PMC7260492

[jcv212164-bib-0008] Brown, T. A. (2006). Confirmatory factor analysis for applied research (1st ed.). Guilford Press.

[jcv212164-bib-0009] Calvo, M. G. , Ramos, P. M. , & Estevez, A. (1992). Test anxiety and comprehension efficiency: The role of prior knowledge and working memory deficits. Anxiety, Stress & Coping, 5(2), 125–138. 10.1080/10615809208250492

[jcv212164-bib-0010] Collishaw, S. (2015). Annual research review: Secular trends in child and adolescent mental health. Journal of Child Psychology and Psychiatry, 56(3), 370–393. 10.1111/jcpp.12372 25496340

[jcv212164-bib-0011] Crawley, E. , Loades, M. , Feder, G. , Logan, S. , Redwood, S. , & Macleod, J. (2020). Wider collateral damage to children in the UK because of the social distancing measures designed to reduce the impact of COVID‐19 in adults. BMJ Paediatrics Open, 4(1), 000701. 10.1136/bmjpo-2020-000701 PMC7223269 32420459

[jcv212164-bib-0012] Daroische, R. , Hemminghyth, M. S. , Eilertsen, T. H. , Breitve, M. H. , & Chwiszczuk, L. J. (2021). Cognitive impairment after COVID‐19—A review on objective test data. Frontiers in Neurology, 1238. 10.3389/fneur.2021.699582 PMC8357992 34393978

[jcv212164-bib-0013] Dewa, L. H. , Crandell, C. , Choong, E. , Jaques, J. , Bottle, A. , Kilkenny, C. , Lawrence‐Jones, A. , Di Simplicio, M. , Nicholls, D. , & Aylin, P. (2021). CCopeY: A mixed‐methods coproduced study on the mental health status and coping strategies of young people during COVID‐19 UK lockdown. The Journal of Adolescent Health: Official Publication of the Society for Adolescent Medicine, 68(4), 202112–202675. 10.1016/j.jadohealth.2021.01.009 PMC9188746 33589305

[jcv212164-bib-0014] Essau, C. A. , Lewinsohn, P. M. , Olaya, B. , & Seeley, J. R. (2014). Anxiety disorders in adolescents and psychosocial outcomes at age 30. Journal of Affective Disorders, 163, 125–132. 10.1016/j.jad.2013.12.033 24456837 PMC4028371

[jcv212164-bib-0015] Evans, I. E. , Martyr, A. , Collins, R. , Brayne, C. , & Clare, L. (2019). Social isolation and cognitive function in later life: A systematic review and meta‐analysis. Journal of Alzheimer's Disease, 70(s1), S119–S144. 10.3233/jad-180501 PMC6700717 30372678

[jcv212164-bib-0016] Eysenck, M. W. , & Calvo, M. G. (1992). Anxiety and performance: The processing efficiency theory. Cognition & Emotion, 6, 409–434. 10.1080/02699939208409696

[jcv212164-bib-0017] Eysenck, M. W. , Derakshan, N. , Santos, R. , & Calvo, M. G. (2007). Anxiety and cognitive performance: Attentional control theory. Emotion, 7(2), 336–353. 10.1037/1528-3542.7.2.336 17516812

[jcv212164-bib-0018] Fancourt, D. , Steptoe, A. , & Bu, F. (2021). Trajectories of anxiety and depressive symptoms during enforced isolation due to Covid‐19 in England: A longitudinal observational study. The Lancet Psychiatry, 8(2), 141–149. 10.1016/s2215-0366(20)30482-x 33308420 PMC7820109

[jcv212164-bib-0019] Ingram, J. , Hand, C. J. , & Maciejewski, G. (2021). Social isolation during COVID‐19 lockdown impairs cognitive function. Applied Cognitive Psychology, 35, 935–947. 10.1002/acp.3821 34230768 PMC8250848

[jcv212164-bib-0020] Kline, T. (2005). Psychological testing: A practical approach to design and evaluation. Sage.

[jcv212164-bib-0021] Kwong, A. S. F. , Pearson, R. M. , Adams, M. J. , Northstone, K. , Tilling, K. , Smith, D. , Fawns‐Ritchie, C. , Bould, H. , Warne, N. , Zammit, S. , Gunnell, D. J. , Moran, P. A. , Micali, N. , Reichenberg, A. , Hickman, M. , Rai, D. , Haworth, S. , Campbell, A. , Altschul, D. , & Timpson, N. J. (2021). Mental health before and during the Covid‐19 pandemic in two longitudinal UK population cohorts. The British Journal of Psychiatry, 218(6), 334–343. 10.1192/bjp.2020.242 33228822 PMC7844173

[jcv212164-bib-0022] McElroy, E. , Patalay, P. , Moltrecht, B. , Shevlin, M. , Shum, A. , Creswell, C. , & Waite, P. (2020). Demographic and health factors associated with pandemic anxiety in the context of COVID‐19. British Journal of Health Psychology, 25(4), 934–944. 10.1111/bjhp.12470 32860334

[jcv212164-bib-0023] Merikangas, K. R. , He, J. , Burstein, M. , Swanson, S. A. , Avenevoli, S. , Cui, L. , Benjet, C. , Georgiades, K. , & Swendsen, J. (2010). Lifetime prevalence of mental disorders in U.S. Adolescents: Results from the national comorbidity survey replication‐adolescent supplement (NCS‐A). Journal of the American Academy of Child and Adolescent Psychiatry, 49(10), 980–989. 10.1016/j.jaac.2010.05.017 20855043 PMC2946114

[jcv212164-bib-0024] Moran, T. P. (2016). Anxiety and working memory capacity: A meta‐analysis and narrative review. Psychological Bulletin, 142(8), 831–864. 10.1037/bul0000051 26963369

[jcv212164-bib-0025] NHS Digital . (2020). Mental health of children and young people in England, 2020: Wave 1 follow up to the 2017 survey. NHS Digital.

[jcv212164-bib-0026] NHS Digital, Mental Health Team . (2021). Mental health services monthly statistics final March provisional April 2021. NHS Digital.

[jcv212164-bib-0027] Orben, A. , Tomova, L. , & Blakemore, S. (2020). The effects of social deprivation on adolescent development and mental health. The Lancet Child & Adolescent Health, 4(8), 634–640. 10.1016/s2352-4642(20)30186-3 32540024 PMC7292584

[jcv212164-bib-0028] Patil, V. H. , Singh, S. N. , Mishra, S. , & Donavan, D. T. (2017). Parallel analysis engine to aid in determining number of factors to retain using R [computer software]. Available from https://analytics.gonzaga.edu/parallelengine/

[jcv212164-bib-0029] Paus, T. , Keshavan, M. , & Giedd, J. N. (2008). Why do many psychiatric disorders emerge during adolescence? Nature Reviews Neuroscience, 9(12), 947–957. 10.1038/nrn2513 19002191 PMC2762785

[jcv212164-bib-0030] Pierce, M. , Hope, H. , Ford, T. , Hatch, S. , Hotopf, M. , John, A. , Kontopantelis, E. , Webb, R. , Wessely, S. , McManus, S. , & Abel, K. M. (2020). Mental health before and during the COVID‐19 pandemic: A longitudinal probability sample survey of the UK population. The Lancet Psychiatry, 7(10), 883–892. 10.1016/s2215-0366(20)30308-4 32707037 PMC7373389

[jcv212164-bib-0031] Polanczyk, G. V. , Salum, G. A. , Sugaya, L. S. , Caye, A. , & Rohde, L. A. (2015). Annual research review: A meta‐analysis of the worldwide prevalence of mental disorders in children and adolescents. The Journal of Child Psychology and Psychiatry and Allied Disciplines, 56(3), 345–365. 10.1111/jcpp.12381 25649325

[jcv212164-bib-0032] Racine, N. , McArthur, B. A. , Cooke, J. E. , Eirich, R. , Zhu, J. , & Madigan, S. (2021). Global prevalence of depressive and anxiety symptoms in children and adolescents during COVID‐19: A meta‐analysis. JAMA Pediatrics, 175(11), 1142–1150. 10.1001/jamapediatrics.2021.2482 34369987 PMC8353576

[jcv212164-bib-0033] Rapee, R. M. , Oar, E. L. , Johnco, C. J. , Forbes, M. K. , Fardouly, J. , Magson, N. R. , & Richardson, C. E. (2019). Adolescent development and risk for the onset of social‐emotional disorders: A review and conceptual model. Behaviour Research and Therapy, 123, 103501. 10.1016/j.brat.2019.103501 31733812

[jcv212164-bib-0034] Ree, M. J. , MacLeod, C. , French, D. , & Locke, V. (2000). The state–trait inventory for cognitive and somatic anxiety: Development and validation. In Poster session presented at the annual meeting of the association for the advancement of behavior therapy.

[jcv212164-bib-0035] Roberts, K. E. , Hart, T. A. , & Eastwood, J. D. (2016). Factor structure and validity of the state‐trait inventory for cognitive and somatic anxiety. Psychological Assessment, 28(2), 134–146. 10.1037/pas0000155 26011481

[jcv212164-bib-0036] Sadler, K. , Vizard, T. , Ford, T. , Goodman, A. , Goodman, R. , & McManus, S. (2018). Mental health of children and young people in England, 2017: Trends and characteristics. NHS Digital.

[jcv212164-bib-0037] Smirni, P. , Lavanco, G. , & Smirni, D. (2020). Anxiety in older adolescents at the time of COVID‐19. Journal of Clinical Medicine, 9(10), 3064. 10.3390/jcm9103064 32977568 PMC7598163

[jcv212164-bib-0038] Soto, E. F. , Kofler, M. J. , Singh, L. J. , Wells, E. L. , Irwin, L. N. , Groves, N. B. , & Miller, C. E. (2020). Executive functioning rating scales: Ecologically valid or construct invalid? Neuropsychology, 34(6), 605–619. 10.1037/neu0000681 32730048 PMC7483691

[jcv212164-bib-0039] Twenge, J. M. , Baumeister, R. F. , Tice, D. M. , & Stucke, T. S. (2001). If you can't join them, beat them: Effects of social exclusion on aggressive behavior. Journal of Personality and Social Psychology, 81(6), 1058–1069. 10.1037/0022-3514.81.6.1058 11761307

[jcv212164-bib-0040] Van Ameringen, M. , Mancini, C. , & Farvolden, P. (2003). The impact of anxiety disorders on educational achievement. Journal of Anxiety Disorders, 17(5), 561–571. 10.1016/s0887-6185(02)00228-1 12941366

[jcv212164-bib-0041] Yong, A. G. , & Pearce, S. (2013). A beginner’s guide to factor analysis: Focusing on exploratory factor analysis. Tutorials in Quantitative Methods for Psychology, 9(2), 79–94. 10.20982/tqmp.09.2.p079

