# Supplementary material for: Investigating the impact of the COVID‐19 pandemic on older adolescents' psychological wellbeing and self‐identified cognitive difficulties
Source: JCPP Adv. 2023 May 29;3(4):e12164. doi: 10.1002/jcv2.12164 (PMC10694530; doi:10.1002/jcv2.12164)
Supplement: Supplementary file 1 — Supporting Information S1 [file JCV2-3-e12164-s001.pdf]

Online Supplement: Investigating the impact of the COVID-19 pandemic on older adolescents' psychological wellbeing and self-identified cognitive difficulties

Meg Attwood[a] and Christopher Jarrold[a]

[a]School of Psychological Science, University of Bristol

This online supplement provides additional supporting information for our manuscript entitled: "Investigating the impact of the COVID-19 pandemic on older adolescents' psychological wellbeing and self-identified cognitive difficulties".

Contextual information and supplementary analyses are presented as follows:

S1. Contextual information relevant to the study

S2. Impacts of the pandemic on overall wellbeing

S3. Impacts of the pandemic on anxiety

S4. Self-identified cognitive difficulties associated with anxiety

S5. A note on sample sizes and sample characteristics

S1. Contextual information relevant to the study

S1.1. Overview of relevant social distancing measures in place during the 12-month study period

On 23 March 2020, the United Kingdom (UK) entered its first national lockdown as part of measures to contain the spread of the COVID-19 virus. At this time, schools and colleges were closed to all except a small minority of children and young people (those identified as vulnerable and children of designated keyworkers), measures which resulted in an unprecedented shift to distance learning and subsequent cancellation of school-leaving GCSE and A-level examinations. Phased reopening of schools in England began on 1 June 2020, but the majority of secondary schools and colleges remained closed until after the summer break. In September 2020 most young people returned to school. A second national lockdown lasting 4 weeks began on 4 November 2020 with students returning to school from 2 December 2020. A further national lockdown began on 6 January 2021 with students returning to school from 8 March 2021. Although there was variability in the extent to which social distancing measures were imposed in different parts of the country over the 12 month period this study covers, surveys were timed to coincide with national-level restrictions (timepoints 1 and 3) and large-scale returns to schooling (timepoint 2).

S1.2. Full survey schedule (timepoint 1)

	**Wellbeing**
W1	**Wellbeing refers to your mood, your sense of purpose and how satisfied you are feeling with your life as a whole. How would you rate your wellbeing (over the past week) on a scale from 0 - 10, where 10 is feeling your very best and 0 is feeling your worst?**
W2	**How has the COVID-19 pandemic affected your overall wellbeing? Please tick one of the options below.**
	My overall wellbeing has stayed the same
	My overall wellbeing has increased
	My overall wellbeing has decreased
W3	**If you could only choose only one of the following, which would you say has contributed most to your decrease in wellbeing?**
	Disruption to daily routines
	School closures
	Reduced social contact
	Relationship difficulties (friends or family)
	Anxiety or worries
	Uncertainty about the future
	Low mood
	Other?
W4	**How have you been feeling over the past week. Select the most appropriate responses below (never, sometimes, most of the time, always).**
	I feel calm
	I have to distract myself from negative thoughts
	I feel upbeat
	I have been feeling worried or anxious
	I am able to get things done
	I feel lonely
	I am coping well
	I feel overwhelmed
	I feel good about my daily routine
	I am lacking motivation
	I feel positive about my future
W5	**I am engaging in physical activity/exercise...**
	The right amount for me
	Less than I'd like
	More than I'd like
W6	**I am sleeping…**
	The right amount for me
	Less than I'd like
	More than I'd like
W7	**I am socialising (within COVID-19 guidance)...**
	The right amount for me
	Less than I'd like
	More than I'd like

	Anxiety
A1	**How anxious have you been feeling over the past week? Please rate your level of anxiety on a scale from 0 - 10 where 10 is the most anxious you could feel and 0 is the least anxious you could feel.**
A2	**How has the COVID-19 pandemic affected your levels of anxiety? Tick the statement below that best describes you.**
	I don't feel any more anxious than usual
	I feel less anxious than I usually do
	I feel more anxious than I usually do
A3	**What has contributed most to your anxiety? If this question is not applicable to you, please click on the blue arrow to skip it.**
	**1. Drag and drop the items that have affected your anxiety into the central box. Select only those items that apply to your situation.**
	**2. Click and drag these items within the box to arrange them in rank order (i.e., item 1 is the item that has most affected your anxiety).**
	School closures/ Returning to school*
	Disruption to my daily routine
	Examination disruption
	University uncertainty
	Limited social contact
	Relationship difficulties (friends or family)
	Worries about my health
	Concerns about family or friends
	News feeds
	Uncertainty about the future
	Other?
A4	**Are you experiencing any of the following concerns? Tick all of the statements that apply to you.**
	I am worried about my physical health
	I am concerned about my psychological wellbeing
	I am worried about the health and wellbeing of family or friends
	I have concerns about my schoolwork
	I am worried about my future (e.g., examinations, university applications)
	I am concerned about my job prospects
	Other concerns not listed above (please specify)
	Not applicable to me
A5	**If you are currently experiencing anxiety, which of these aspects of your life have been affected most? If this question is not applicable to you, please click on the blue arrow to skip it.**
	**1. Drag and drop the areas of your life that have been affected by anxiety into the central box. Select only those items that apply to your situation.**
	**2. Click and drag these items around within the box to arrange them in rank order (item 1 is the area of your life most affected by anxiety).**
	Overall wellbeing
	Sleep
	Eating habits
	Exercise
	Relationships with family or friends
	Mood
	Schoolwork
	Focus and concentration
	Motivation
	Other?
A6	**Trait STICSA measure (Ree et al., 2000); timepoints 2 & 3 also included Pandemic Anxiety Scale (McElroy et al., 2020)**

	Cognition
C1	**Over the past week, how would you describe your ability to focus and think clearly (in general)? (never, sometimes, most of the time, always).**
	I struggle to remember important things
	I am able to focus when I need to
	My mind wanders when I should be concentrating
	I can think clearly
	I struggle to focus on one task at a time
C2	**Over the past week, how would you describe your ability to plan and organise your day, and complete tasks (in general)? (never, sometimes, most of the time, always).**
	I feel motivated
	I am able to structure my day
	I feel overwhelmed by the amount I have to do in a day
	I am able to prioritise my to do list
	I struggle to work on one task at a time
	I can complete important tasks within the time allowed

	School experience
S1	**Please select the most appropriate option below.**<br>I am not currently attending school. I am doing all of my learning from home.<br>I am currently attending school. I am the child of a keyworker.<br>I am currently attending school. I am not the child of a keyworker.<br>I am in Year 13 and currently have no schoolwork to complete.<br>Other?
S2	**How much contact are you having with your teachers to support your learning? This could include email, telephone or videoconferencing e.g., Zoom, and involve one-to-one or group communication. Choose the statement below that best describes your situation and please use the text box to provide a brief description.**<br>I have daily contact with at least one of my teachers.<br>I have weekly contact with at least one of my teachers.<br>I have fortnightly contact with at least one of my teachers.<br>I have irregular contact with my teachers.<br>I have had no contact with my teachers.<br>Other?
S3	**How do you feel your ability to complete your schoolwork has been affected by the COVID-19 pandemic? Please select the most appropriate responses below. (never, sometimes, most of the time, always).**<br>I am able to focus on my schoolwork<br>I worry that I am falling behind<br>I am able to complete challenging work within the time allowed<br>It is easy for me to get distracted<br>I get to the end of the day and I have accomplished what I intended to do<br>Worries distract me from my schoolwork<br>I struggle to remember what I've learnt<br>Other?<br>Not applicable. I am in Year 13 and don't currently have any work set.
S4	**How do you feel (in general) about your schoolwork at the moment? Please select the most appropriate responses below. (never, sometimes, most of the time, always).**<br>I feel motivated to complete my coursework<br>I have been able to access online learning materials<br>I am working at my usual standard<br>Distance learning has been challenging<br>about<br>Other?<br>Not applicable. I am in Year 13 and don't currently have any work set.
	**Intervention**
I1	**During the COVID-19 pandemic, have you identified any strategies or activities that have reduced your anxiety and/or improved your overall level of wellbeing? Please specify below. If this question does not apply to you, then please click the blue arrow to skip it.**

S1.3. Sample characteristics (timepoints 1, 2 and 3) and preliminary screening measures

Table S1.3. Descriptive statistics for Studies 1, 2, and 3.

	**Study 1**		**Study 2**		**Study 3**	
	N	%	N	%	N	%
Gender						
Female	392	64.6	172	71.1	465	75.2
Male	203	33.4	64	26.4	123	19.9
Other (other, prefer not to say)	12	2.0	6	2.5	30	4.9
Year group						
Year 11	90	14.9	5	2.1	74	12.0
Year 12	356	58.8	83	34.6	319	51.7
Year 13	159	26.3	152	63.3	224	36.3
Location						
England	596	98.2	213	88.0	609	98.5
Wales	11	1.8	17	7.0	9	1.5
Scotland	0	0	12	5.0	0	0.0
School attendance*						
Learning from home/ Attending on a full timetable	339	55.8	141	58.8	15	2.4
Attending school (child of a keyworker)/ Attending on a reduced timetable	15	2.5	61	25.4	31	5.0
Attending school (not child of a keyworker)/ Not attending school	35	5.8	10	4.2	23	3.7
No schoolwork set (Y13)/ School not open	156	25.7	20	8.3	549	88.8
Other	62	10.2	8	3.3	0	0.0
Reported change in wellbeing						
Decrease in wellbeing	375	62.1	141	58.3	468	75.7
No change in wellbeing	132	21.9	61	25.2	89	14.4
Increase in wellbeing	97	16.1	40	16.5	61	9.9
Reported change in anxiety						
Decrease in anxiety	77	12.7	19	7.9	47	7.6
No change in anxiety	181	29.8	70	28.9	134	21.8
Increase in anxiety	349	57.5	153	63.2	435	70.6

**The wording of the school attendance item was changed at timepoint 2 to account for the changing context.*

Preliminary screening measures

At each timepoint, participants were excluded if they completed less than 75% of the survey and failed to provide any demographic information, had more than one missing item on the STICSA trait measure or failed to report wellbeing or anxiety (VAS) scores. Single missing items were replaced using mean item substitution. Preliminary checks identified duplicate entries to the survey and these cases were excluded. Mahalanobis Distance scores (computed for wellbeing and anxiety VAS scores and STICSA trait anxiety scores) were used to identify multivariate outliers, and these cases were removed from subsequent analyses. 1 case was removed on these grounds at timepoints 1 and 2, and 2 cases removed at timepoint 3.

S1.4. Correlations between wellbeing and anxiety measures at timepoints 2 and 3

Table S1.4. Pearson Correlations Among Wellbeing and Anxiety Measures (Studies 2 and 3).

**Study 2**	1	*2*	3	4	5	6
1. Wellbeing (VAS)	-					
2. State anxiety (VAS)	-.471**	-				
3. Trait anxiety (STICSA total)	-.506**	.558**	-			
4. Trait Cognitive (STICSA)	-.468**	.500**	.929**	-		
5. Trait Somatic (STICSA)	-.464**	.529**	.910**	.692**	-	
6. Pandemic anxiety (PAS)	-.179**	.302**	.365**	.340**	.331**	-
**Study 3**	1	*2*	3	4	5	6
1. Wellbeing (VAS)	-					
2. State anxiety (VAS)	-.499**	-				
3. Trait anxiety (STICSA total)	-.529**	.592**	-			
4. Trait Cognitive (STICSA)	-.522**	.580**	.914**	-		
5. Trait Somatic (STICSA)	-.427**	.482**	.891**	.629**	-	
6. Pandemic anxiety (PAS)	-.240**	.347**	.434**	.417**	.363**	-

*** Correlation is significant at the 0.01 level (2-tailed).*

S1.5. Gender differences in wellbeing (VAS) and anxiety scores

At all three timepoints, boys reported significantly higher wellbeing scores than girls ($t(593) = 5.362$, $p < .001$, $d = .464$; $t(234) = 2.480$, $p = .014$, $d = .363$; $t(586) = 4.243$, $p < .001$, $d = .430$ respectively). At all three timepoints, boys had significantly lower state ($t(593) = -6.257$, $p < .001$, $d = -.541$; $t(234) = -4.262$, $p < .001$, $d = -.624$; $t(177.110) = -3.149$, $p = .002$, $d = -.341$ respectively) and trait anxiety scores ($t(471.239) = -7.917$, $p < .001$, $d = -.650$; $t(234) = -7.211$, $p < .001$, $d = -1.056$; $t(586) = -7.689$, $p < .001$, $d = -.780$ respectively) than girls. Boys also had significantly lower PAS scores than girls at timepoints 2 and 3 ($t(234) = -4.308$, $p < .001$, $d = -.631$; $t(584) = -8.386$, $p < .001$, $d = -.853$ respectively).

S2. Wellbeing impacts

S2.1. Gender differences in reporting of pandemic-related wellbeing impacts

Supplementary analyses sought to establish the extent of any gender differences in reporting of pandemic-related impacts on wellbeing. At timepoint 1, the distribution of respondents across the three impact categories (increase, no change, decrease in wellbeing) differed significantly for males and females (X^2 $(2, N = 592) = 12.661$, $p = .002$). Bonferroni-adjusted post hoc tests using cellwise residual analysis[1] identified significant deviations from the null distribution at timepoint 1. Girls reported greater incidence of decreased wellbeing (X^2 $(1, N = 259) = 12.46$, $p < .001$) and lesser incidence of no change

[1] Beasley, T.M. and Schumacker, R.E. (1995) Multiple Regression Approach to Analyzing Contingency Tables: Post Hoc and Planned Comparison Procedures. *The Journal of Experimental Education*, 64, 79-93.
Garcia-Perez, M. A., & Nunez-Anton, V. (2003). Cellwise residual analysis in two-way contingency tables. *Educational and psychological measurement*, *63*, 825-839.

in wellbeing (X^2 (1, N = 73) = 7.45, p = .006) than expected. At timepoints 2 and 3, the distribution of respondents across impact groups again differed significantly by gender (X^2 (2, N = 236) = 13.205, p = .001; X^2 (2, N = 588) = 11.227, p = .004 respectively). Bonferroni-adjusted post hoc tests using cellwise residual analysis identified significant deviations from the null distribution at each timepoint. At both timepoints, girls reported greater incidence of decreased wellbeing (X^2 (1, N = 112) = 13.03, p < .001; X^2 (1, N = 362) = 8.53, p = .004 respectively) and lesser incidence of no change in wellbeing (X^2 (1, N = 36) = 8.01, p = .005; X^2 (1, N = 56) = 10.43, p = .001 respectively) than expected.

Assessing the extent to which wellbeing (VAS) scores differed across pandemic-related wellbeing impact groups.

A further set of supplementary analyses sought to establish whether participants' reporting of pandemic-related impacts on their wellbeing (decrease, no change, increase in wellbeing) were supported by meaningful differences in their wellbeing (VAS) scores. At timepoint 1, there was a significant difference in reported wellbeing scores across the wellbeing impact groups (increase in wellbeing, no change, decrease in wellbeing), (F(2,601) = 71.429, p < .001, η^2 = .192), with Tukey post hoc tests demonstrating significantly lower wellbeing scores for the decreased wellbeing group relative to the increased and no change groups (p < .001, 95% C.I. = [-2.15, -1.23]; p < .001, 95% C.I. = [-2.13, -1.31] respectively), but no significant difference between the no change and increased wellbeing groups (p = .987). At timepoint 2, there was a small, but significant difference in wellbeing scores across the three groups, (F(2,239) = 7.442, p < .001, η^2 = .059), with Tukey post hoc tests demonstrating significantly lower wellbeing scores for the

decreased wellbeing group relative to the increased and no change groups ($p = .006$, 95% C.I. = [-1.88, -.25]; $p = .007$, 95% C.I. = [-1.59, -.20] respectively), but no significant difference between the no change and increased wellbeing groups ($p = .904$). At timepoint 3, the difference in wellbeing scores across impact groups was more marked ($F(2,615) = 70.716$, $p < .001$, $\eta^2 = .187$). Tukey post hoc tests demonstrated significantly lower wellbeing scores for the decreased wellbeing group relative to the increased and no change groups ($p < .001$, 95% C.I. = [-2.98, -1.85]; $p < .001$, 95% C.I. = [-2.08, -1.11] respectively), as well as a significant difference between the no change and increased wellbeing groups ($p = .016$, 95% C.I. = [-1.51, -.13]).

S2.2. Broader wellbeing impacts of the pandemic

Low mood, loneliness, feelings of negativity about the future and difficulty coping were common features of young people's reporting of their overall wellbeing during the pandemic. Across the three timepoints, the majority of young people reported low mood (71%, 72%, 81% reporting feeling upbeat never or only some of the time) and sustained feelings of loneliness were common (31%, 27%, 43% reporting feeling lonely most or all of the time). A striking level of negativity about the future was reported at all three timepoints (61%, 57%, 68% reporting feeling positive about the future never or only some of the time), as was a high degree of difficulty coping with their situation (46%, 48%, 59% reporting feeling they could cope with their situation never or only some of the time).

Related aspects of wellbeing including sleep, opportunities for social interaction, and exercise were also disrupted during the pandemic. At all three timepoints, sleep disturbances were common (55%, 55%, 65% experienced disruptions to their sleep (of

these, 40%, 50%, 57% sleeping less than they would like) respectively). Participants reported limitations on their social interaction (60%, 48%, 72% reporting socialising less than they would like within COVID-19 regulations) and exercise (66%, 60%, 71% reporting exercising less than they would like) at each of the timepoints. These descriptive statistics suggest that detrimental impacts of the pandemic on young people's wellbeing were not limited to periods of lockdown and that young people experienced the greatest disruption to sleep, social interaction and exercise at timepoint 3 during the third UK national lockdown - effects perhaps exacerbated by wintery weather conditions at that time.

S3. Anxiety impacts

S3.1. Supplementary analyses relating to reporting of pandemic-related anxiety impacts

At all three timepoints, the distribution of respondents across the three pandemic-related anxiety impact categories (decrease, no change, increase in anxiety) differed significantly for males and females (X^2 (2, N = 595) = 27.142, $p < .001$; X^2 (2, N = 236) = 14.432, $p < .001$; X^2 (2, N = 586) = 12.259, $p = .002$ respectively). Bonferroni-adjusted post hoc tests identified significant deviations from the null distribution for the increased and no change anxiety groups. At timepoints 1 and 2, girls reported greater incidence of increased anxiety (X^2 (1, N = 252) = 26.32, $p < .001$; X^2 (1, N = 121) = 14.21, $p < .001$ respectively) than expected. At all three timepoints, girls reported lesser incidence of no change in anxiety (X^2 (1, N = 93) = 19.98, $p < .001$; X^2 (1, N = 39) = 11.63, $p < .001$; X^2 (1, N = 88) = 11.63, $p < .001$ respectively) than expected.

Subsequent analyses sought to establish whether participants' reporting of pandemic-related impacts on their anxiety (decrease, no change, increase in anxiety) were supported by meaningful differences in their state (VAS) anxiety scores. At all three timepoints, there was a significant difference between anxiety impact groups in reported state anxiety (VAS) scores ($F(2,604) = 116.279$, $p < .001$, $\eta^2 = .279$); $F(2,239) = 31.450$, $p < .001$, $\eta^2 = .208$); $F(2,613) = 93.87$, $p < .001$, $\eta^2 = .234$ respectively). In all three cases, state anxiety scores were significantly higher in the increased anxiety group relative to both the no change ($p < .001$, 95% C.I. = [2.21, 3.08]; $p < .001$, 95% C.I. = [1.74, 3.30]; $p < .001$, 95% C.I. = [1.91, 2.81] respectively) and decreased anxiety groups ($p = < .001$, 95% C.I. = [1.76, 2.97]; $p = < .001$, 95% C.I. = [.72, 3.35]; $p = < .001$, 95% C.I. = [1.68, 3.09] respectively), but did not differ significantly between the no change and decreased groups ($p = .564$; $p = .695$; $p = .996$).

A series of follow-up logistic regression analyses sought to identify significant predictors of increased anxiety reporting (as compared to reporting of no change in anxiety). Results of these analyses are presented in the tables that follow.

Table S3.1.1. Logistic regression analyses identifying significant predictors of increased anxiety reporting.

Study 1

	B	Sig.	SE	Exp (β)	95% CI
Variable					
Age	-0.088	0.736	0.262	0.916	[.55, 1.53]
State anxiety**	0.504	<.001	0.069	1.655	[1.45, 1.90]
Trait anxiety	0.016	0.240	0.014	1.016	[.99, 1.04]
Gender (base = male)*	0.538	0.035	0.256	1.712	[1.04, 2.83]
School attendance (base = learning from home)		0.188			
Attending school (KW)	0.151	0.812	0.635	1.163	[.34, 4.04]
Attending school (not KW)	-1.528	0.094	0.912	0.217	[.04, 1.30]
Y13 (no schoolwork)	-0.338	0.663	0.774	0.714	[.16, 3.25]
Other	-0.234	0.725	0.666	0.791	[.22, 2.92]
Teacher contact (base = no contact)		0.603			
Irregular	0.353	0.276	0.324	1.424	[.75, 2.69]
Fortnightly	-0.218	0.661	0.497	0.804	[.30, 2.13]
Weekly	0.125	0.743	0.381	1.133	[.54, 2.39]
Daily	0.562	0.288	0.529	1.754	[.62, 4.94]
Missing	-0.332	0.662	0.760	0.717	[.16, 3.18]
Challenge accessing online learning (base = never)		0.124			
Some of the time	0.642	0.038	0.310	1.901	[1.04, 3.49]
Most of the time	0.733	0.119	0.470	2.082	[.83, 5.23]
Always	0.354	0.743	1.080	1.425	[.17, 11.83]
Missing	-0.953	0.388	1.104	0.385	[.04, 3.35]
Distance learning challenging (base = never)*		0.022			
Some of the time	0.392	0.499	0.578	1.479	[.48, 4.60]
Most of the time*	1.213	0.041	0.595	3.363	[1.05, 10.79]
Always	1.153	0.054	0.599	3.169	[.98, 10.24]
Missing	1.869	0.088	1.094	6.483	[.76, 55.34]
Constant	-0.668	0.417	0.823	0.513	
-2LL	467.719				
	X^2 (21, N = 516) = 195.878, p <.001				
Nagelkerke R2	44%				
Hosmer & Lemeshow test	p = .681				
Classification accuracy	78.10%				

All continuous variables were centred at the mean.

Table S3.1.2. Logistic regression analyses identifying significant predictors of increased anxiety reporting.

Study 2

	B	Sig.	SE	Exp (β)	95% CI
Variable					
Age*	0.857	0.020	0.369	2.355	[1.14, 4.85]
State anxiety**	0.435	<.001	0.099	1.544	[1.27, 1.87]
Trait anxiety	0.013	0.542	0.021	1.013	[.97, 1.05]
Gender (base = male)	0.227	0.615	0.452	1.255	[.52, 3.04]
School attendance (base = school not open)		0.365			
Not attending school	-1.406	0.220	1.148	0.245	[.03, 2.32]
Attending on a reduced timetable	-1.517	0.089	0.892	0.219	[.04, 1.26]
Attending on a full timetable	-0.843	0.346	0.895	0.430	[.08, 2.49]
Other	-0.803	0.602	1.540	0.448	[.02, 9.17]
Challenge accessing online learning (base = never)		0.519			
Some of the time	-0.116	0.795	0.446	0.891	[.37, 2.14]
Most of the time	0.758	0.231	0.633	2.134	[.62, 7.38]
Always	-0.426	0.653	0.857	0.653	[.12, 3.50]
Distance learning challenging (base = never)		0.096			
Some of the time	0.865	0.140	0.587	2.376	[.75, 2.50]
Most of the time	1.431	0.018	0.606	4.181	[1.27, 13.72]
Always	1.257	0.035	0.596	3.514	[1.09, 11.29]
Constant	0.821	0.423	1.024	2.272	
-2LL	187.775				
	X^2 (14, N = 210) = 75.208, p <.001				
Nagelkerke R2	42%				
Hosmer & Lemeshow test	p = .071				
Classification accuracy	81.00%				

All continuous variables were centred at the mean.

Table S3.1.3. Logistic regression analyses identifying significant predictors of increased anxiety reporting.

Study 3

	B	Sig.	SE	Exp (β)	95% CI
Variable					
Age	0.089	0.599	0.170	1.093	[.78, 1.52]
State anxiety**	0.510	<.001	0.071	1.666	[1.45, 1.93]
Trait anxiety	0.006	0.654	0.014	1.006	[.98, 1.03]
Gender (base = male)					
Female	0.079	0.796	0.304	1.082	[1.45, 1.91]
School attendance (base = school not open)		0.205			
Not attending school	0.193	0.763	0.640	1.212	[.35, 4.25]
Attending on a reduced timetable	-0.835	0.084	0.482	0.434	[.38, 4.76]
Challenge accessing online learning (base = never)		0.881			
Some of the time	-0.037	0.890	0.264	0.964	[.58, 1.62]
Most of the time	0.295	0.647	0.645	1.343	[.38, 4.76]
Distance learning challenging (base = never)**		<.001			
Some of the time	1.054	0.050	0.539	2.870	[1.00, 8.25]
Most of the time*	1.730	0.001	0.539	5.640	[1.96, 16.21]
Always**	2.003	<.001	0.538	7.408	[2.58, 21.25]
Constant	-0.083	0.878	0.541	0.920	

-2LL	436.681
	X^2 (11, N = 526) = 147.080, p <.001
Nagelkerke R2	36%
Hosmer & Lemeshow test	p = .111
Classification accuracy	83.70%

All continuous variables were centred at the mean.

S3.2. Predominant contributors to anxiety at each timepoint

The figures below present proportional ranked responses (to survey question A3) and highlight predominant contributors to anxiety in older adolescents at each of the three timepoints.

Figure S3.2.1. Contributors to anxiety (timepoint 1).

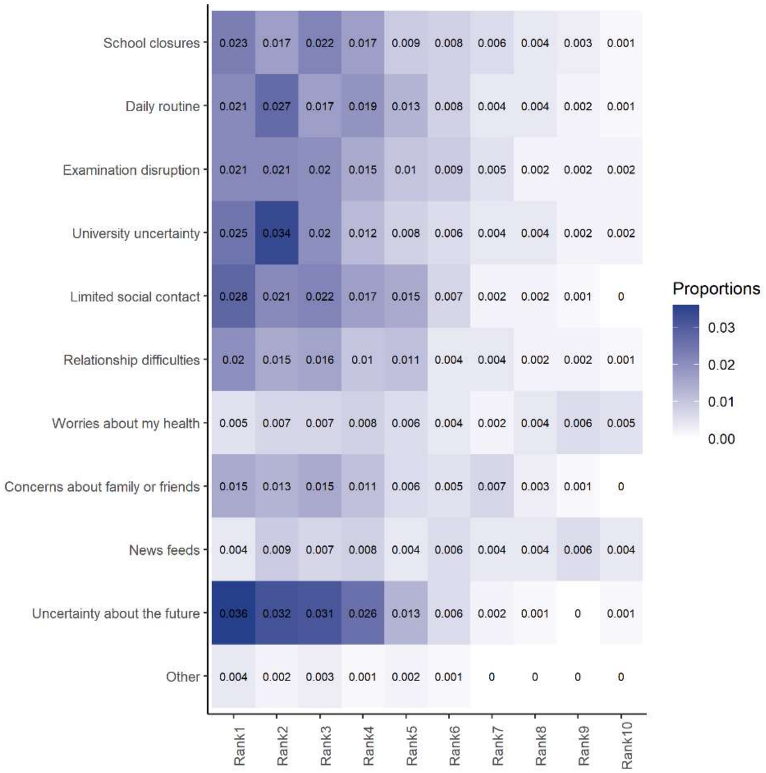

Figure S3.2.2. Contributors to anxiety (timepoint 2).

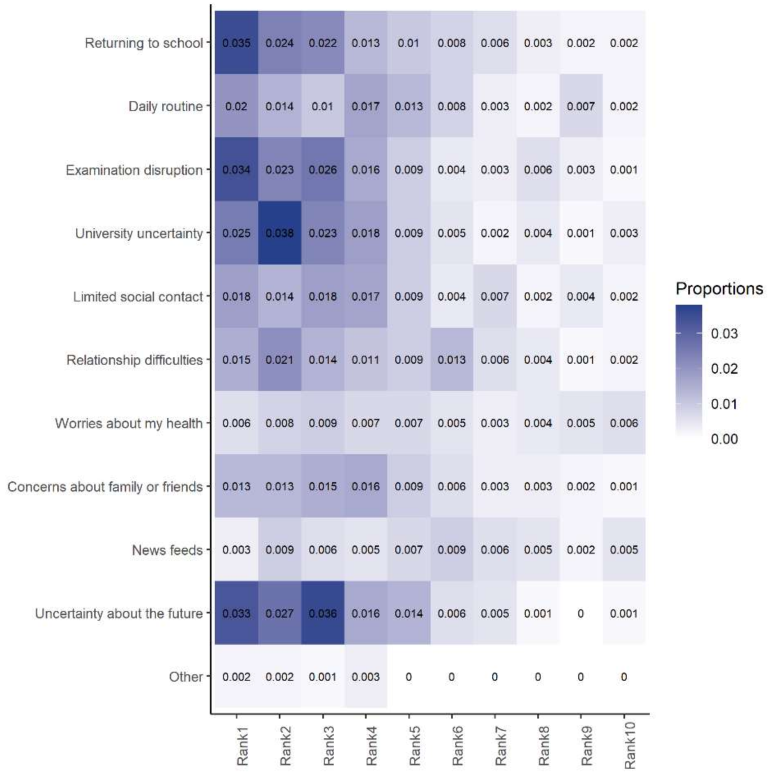

Figure S3.2.3. Contributors to anxiety (timepoint 3).

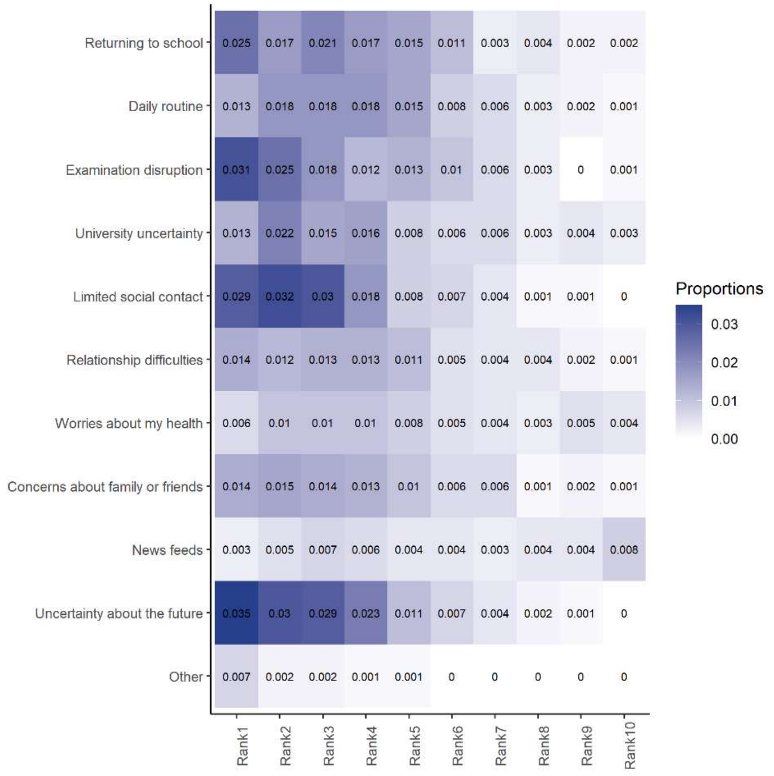

S3.3. Nature of young people's concerns during the pandemic

The figures presented below identify the nature and extent of young people's concerns during the pandemic, as reported at each of the three timepoints.

Figure S3.3.1. Nature of young people's concerns by gender (timepoint 1).

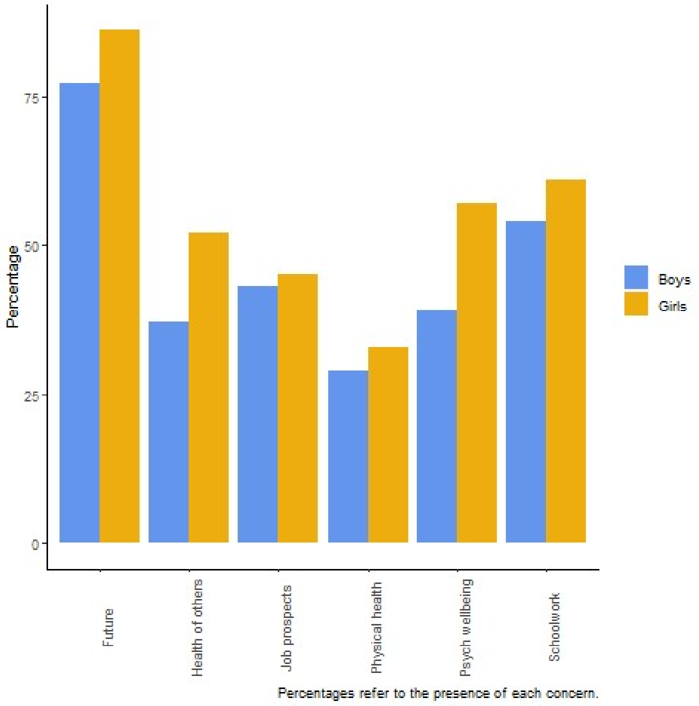

Figure S3.3.2. Nature of young people's concerns by gender (timepoint 2).

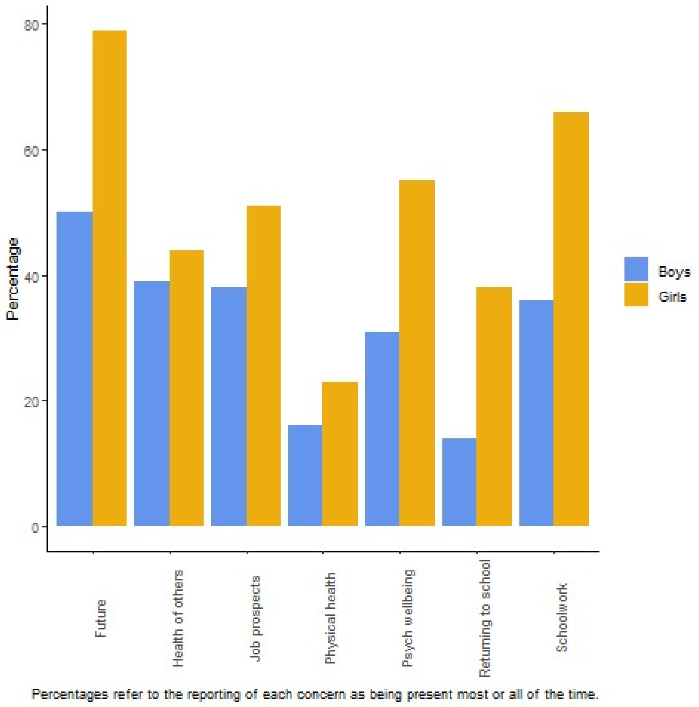

Figure S3.3.3. Nature of young people's concerns by gender (timepoint 3).

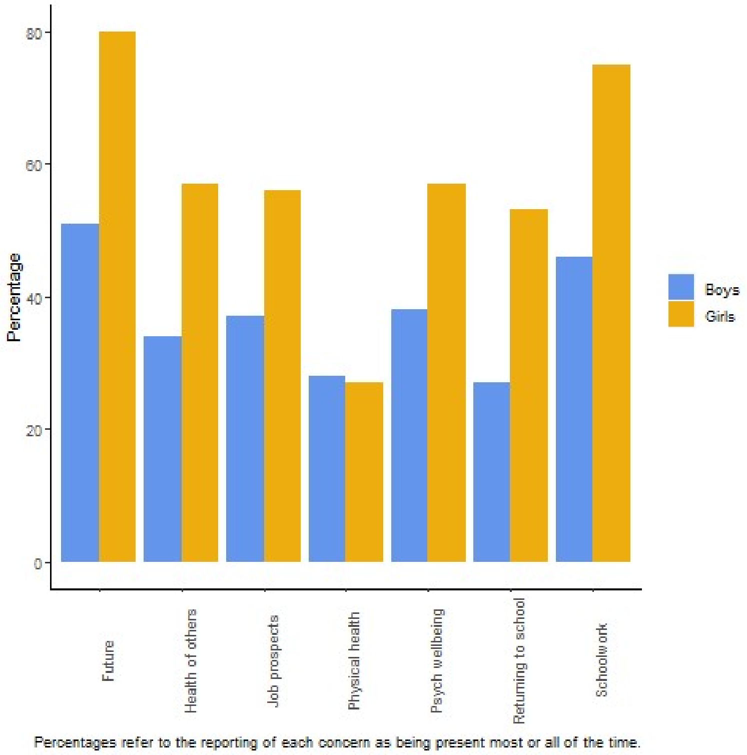

S4. Associations between mood and cognitive difficulties

S4.1. Domains of functioning most affected by anxiety during the pandemic

The figures below present proportional ranked responses (to survey question A5) and highlight the domains of functioning most affected by anxiety at each of the three timepoints.

Figure S4.1.1. Impact of anxiety on functioning (timepoint 1).

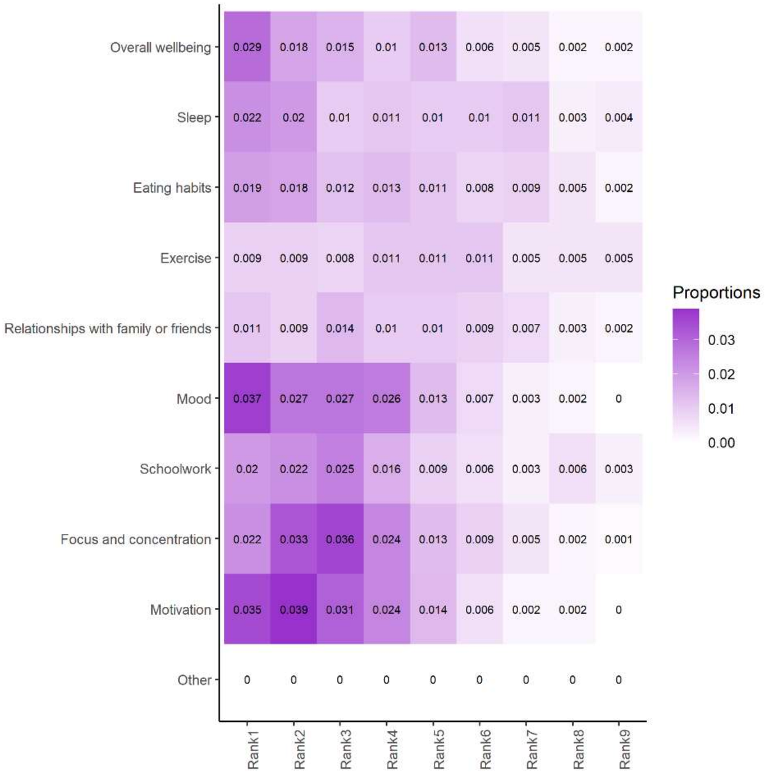

Figure S4.1.2. Impact of anxiety on functioning (timepoint 2).

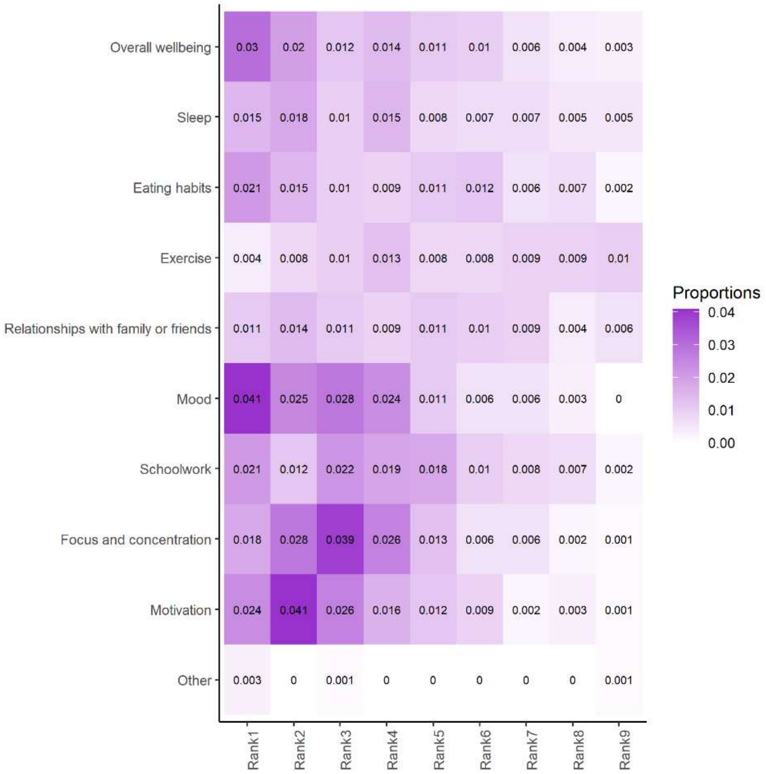

Figure S4.1.3. Impact of anxiety on functioning (timepoint 3).

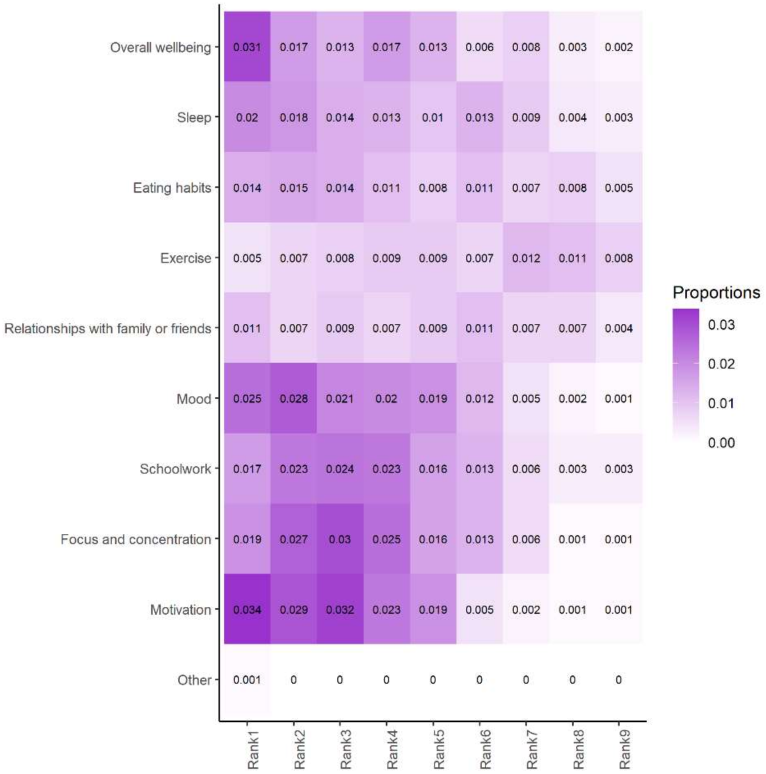

S4.2. Logistic regression analyses relating mood and cognitive difficulties to likelihood of reporting pandemic-related concerns (timepoint 1)

The table presented below supplements findings reported in Figure 1. This series of logistic regression analyses assesses the likelihood of young people reporting the presence of each worry, as predicted by EFA factor scores at timepoint 1.

Table S4.2. Logistic regression results demonstrating likelihood of worries as predicted by self-identified mood and cognitive difficulties

	**Psychological wellbeing**				**Schoolwork**				**Future**				**Jobs**				**Health of others**				**Physical health**			
Variable	B	SE	Exp (β)	95% CI	B	SE	Exp (β)	95% CI	B	SE	Exp (β)	95% CI	B	SE	Exp (β)	95% CI	B	SE	Exp (β)	95% CI	B	SE	Exp (β)	95% CI
Factor 1	-0.183	0.148	0.833	[.62, 1.11]	-0.035	0.128	0.965	[.75, 1.24]	-0.084	0.160	0.920	[.67, 1.26]	-0.229	0.122	0.795	[.63, 1.01]	0.119	0.121	1.127	[.89, 1.43]	-0.230	0.131	0.794	[.61, 1.03]
Factor 2	1.778 **	0.170	5.917	[4.24, 8.26]	0.482 **	0.125	1.620	[1.27, 2.07]	0.918 **	0.172	2.504	[1.79, 3.51]	0.232 *	0.113	1.261	[1.01, 1.57]	0.513 **	0.116	1.671	[1.33, 2.10]	0.192	0.120	1.212	[.96, 1.53]
Factor 3	-0.088	0.145	0.915	[.69, 1.22]	0.653 **	0.133	1.921	[1.48, 2.50]	0.007	0.169	1.007	[.72, 1.40]	0.010	0.119	1.010	[.80, 1.28]	-0.033	0.120	0.968	[.77, 1.22]	0.087	0.127	1.091	[.85, 1.40]
(Constant)	0.114	0.102	1.121		0.425 **	0.091	1.529		1.821 **	0.131	6.176		-0.241 **	0.083	0.786		-0.137	0.083	0.872		-0.798 **	0.090	0.450	
-2LL	591.022				721.489				498.335				810.587				813.058				736.344			
	X^2 (3, N = 606) = 248.538, p <.001				X^2 (3, N = 606) = 100.669, p <.001				X^2 (3, N = 606) = 57.296, p <.001				X^2 (3, N = 606) = 21.403, p <.001				X^2 (3, N = 606) = 24.395, p <.001				X^2 (3, N = 606) = 20.500, p <.001			
Nagelkerke R^2	45%				21%				15%				5%				5%				5%			
Hosmer & Lemeshow test	p = .620				p = .204				p = .138				p = .454				p = .257				p = .200			
Classification accuracy	77.20%				69.30%				84.00%				59.40%				57.80%				68.30%			

S4.3. Model fit indices for a two-factor CFA model for timepoint 2

Table S4.3.1. Alternative Two-Factor Model Fit Indices (CFA, timepoint 2).

**CFA Model**	X^2 (*df*)	*p*	CMIN/DF	TLI	CFI	RMSEA (90% CI)	SRMR
Timepoint 2 (N = 234)							
2-factor model	424.02 (187)	**	2.32	.88	.90	.075 [.066, .085]	.0569

Abbreviations: *CMIN/DF, chi-square fit statistics/degrees of freedom; TLI, Tucker-Lewis index; CFI, comparative fit index; RMSEA, root mean square of approximation; SRMR, standardized root mean square residual.*

The table below presents results from a series of ordinal regression analyses to assess the likelihood of young people reporting increasing frequency of pandemic-related concerns, as predicted by factor scores from timepoint 2 (using the above 2-factor CFA model).

Table S4.3.2. Ordinal regression results demonstrating extent of worries as predicted by self-identified mood and cognitive difficulties

	Returning to school				Psychological wellbeing				Physical health				Schoolwork			
Variable	B	SE	Exp (β)	95% CI	β	SE	Exp (β)	95% CI	B	SE	Exp (β)	95% CI	β	SE	Exp (β)	95% CI
Factor 1	-.862	.442	.422	[.18, 1.01]	.235	.458	1.265	[.52, 3.10]	-.968 *	.440	.380	[.16, .90]	-2.027 **	.480	.132	[.05, .34]
Factor 2	1.297 **	.383	3.660	[1.73, 7.75]	3.061 **	.438	21.350	[9.05, 50.38]	.130	.371	1.138	[.55, 2.35]	1.364 **	.397	3.913	[1.80, 8.52]
-2LL	521.992<br>X^2 (2, N = 234) = 80.696, *p* <.001				142.162<br>X^2 (2, N = 234) = 142.162, *p* <.001				542.151<br>X^2 (2, N = 234) = 20.606, *p* <.001				452.016<br>X^2 (2, N = 234) = 146.485, *p* <.001			
R^2 (Cox and Snell)	32%				46%				8%				47%			
Goodness of fit	*p* = .933				*p* = .134				*p* = .734				*p* = .994			
Test of parallel lines	*p* = .083				*p* = .798				*p* = .019				*p* = .268			

	Jobs				Health of others				Future			
Variable	β	SE	Exp (β)	95% CI	β	SE	Exp (β)	95% CI	β	SE	Exp (β)	95% CI
Factor 1	-.503	.369	.605	[.26, 1.40]	-.070	0.444	.933	[.39, 2.23]	-.702	.466	.496	[.20, 1.23]
Factor 2	0.232 *	.429	2.821	[1.37, 5.82]	.526	0.380	1.691	[.80, 3.56]	2.009 **	.414	7.455	[3.31, 16.78]
-2LL	578.746<br>X^2 (2, N = 234) = 48.066, *p* <.001				516.547<br>X^2 (2, N = 234) = 8.203, p = .017				440.797<br>X^2 (2, N = 234) = 115.681, *p* < .001			
R^2 (Cox and Snell)	19%				3%				39%			
Goodness of fit	*p* = .342				*p* = .416				*p* < .001			
Test of parallel lines	*p* = .243				*p* = .804				*p* < .001			

S4.4. Ordinal regression analyses to examine the extent to which mood and cognitive difficulties at timepoint 3 predicted young people's reporting of pandemic-related concerns

This series of ordinal regression analyses assesses the likelihood of young people reporting increasing frequency of pandemic-related worries at timepoint 3, as predicted by CFA factor scores from timepoint 3. Two models (relating to worries about psychological wellbeing, and schoolwork) failed to meet all necessary assumptions for ordinal regression; results for these models should therefore be interpreted with caution. As at timepoint 1, increased mood difficulties (factor 2) predicted all concerns, but with varying strength. The ability to plan and prioritise (factor 1) predicted the likelihood of concerns regarding the health of others, difficulties with focus and concentration (factor 3) predicted concerns regarding jobs, and both factors 1 and 3 predicted worries about schoolwork (although in all cases, mood difficulties were the stronger predictor).

Table S4.4. Ordinal regression results demonstrating extent of worries as predicted by self-identified mood and cognitive difficulties (timepoint 3).

	**Returning to school**				**Psychological wellbeing**				**Physical health**				**Schoolwork**			
Variable	B	SE	Exp (β)	95% CI	β	SE	Exp (β)	95% CI	B	SE	Exp (β)	95% CI	B	SE	Exp (β)	95% CI
Factor 1	-.126	.229	.882	[.56, 1.38]	.149	.250	1.160	[.71, 1.89]	-.138	.233	.871	[.55, 1.38]	-1.744 **	.255	.175	[.11, .29]
Factor 2	1.330 **	.225	3.779	[2.43, 5.88]	4.459 **	.306	86.405	[47.42, 157.43]	1.220 **	.229	3.388	[2.16, 5.30]	.762 *	.238	2.142	[1.34, 3.42]
Factor 3	.262	.202	1.300	[.88, 1.93]	-.254	.219	.776	[.51, 1.19]	-.079	.205	.924	[.62, 1.38]	.580 *	.217	1.786	[1.17, 2.73]
-2LL	1498.916				1105.295				1419.416				1151.216			
	X^2 (3, N = 609) = 131.727, *p* <.001				X^2 (3, N = 609) = 469.201, *p* <.001				X^2 (3, N = 609) = 70.971, *p* <.001				X^2 (3, N = 609) = 342.659, *p* <.001			
R^2 (Cox and Snell)	20%				54%				11%				43%			
Goodness of fit	*p* = .405				*p* = .996				*p* = .777				*p* < .001			
Test of parallel lines	*p* = .637				*p* = .048				*p* = .505				*p* = .080			

	**Jobs**				**Health of others**				**Future**			
Variable	B	SE	Exp (β)	95% CI	B	SE	Exp (β)	95% CI	β	SE	Exp (β)	95% CI
Factor 1	-.039	.228	.962	[.62, 1.50]	.827 **	.238	2.285	[1.43, 3.65]	*-.366*	.238	.694	[.44, 1.11]
Factor 2	1.053 **	.222	2.865	[1.86, 4.43]	1.916 **	.239	6.797	[4.25, 10.87]	1.399 **	.235	4.050	[2.56, 6.41]
Factor 3	.494 *	.202	1.639	[1.10, 2.43]	.323	.207	1.381	[.92, 2.07]	.332	.211	1.394	[.92, 2.11]
-2LL	1511.124				1352.208				1254.486			
	X^2 (3, N = 609) = 119.632, *p* <.001				X^2 (3, N = 609) = 113.771, *p* = .001				X^2 (3, N = 609) = 172.041, *p* < .001			
R^2 (Cox and Snell)	18%				11%				25%			
Goodness of fit	*p* = .384				*p* = .100				*p* = .059			
Test of parallel lines	*p* = .061				*p* = .838				*p* = .641			

S5. Note on sample size and sample characteristics

Despite the use of a consistent recruitment strategy, sample size differs across timepoints. The reduced sample size at timepoint 2 is likely explained by young people's return to school. While we cannot be certain, schools/colleges may not have prioritised distribution of our survey at this timepoint, on account of academic pressures (catching up on "lost learning") and administrative challenges (relating to staffing and school attendance). Where the survey was distributed by schools, young people may have had less free time available to complete the survey, or reduced motivation to participate in the study given the relaxing of social distancing measures and the reopening of schools.

Though the demographics of the samples (i.e., age and gender distribution) were statistically significantly different across timepoints (see results of post-hoc analyses below), we would argue that these are not likely to be meaningful differences (note the small effect sizes and the small "real" difference between mean ages across timepoints - 3.8 to 4.8 months).

Results of a one-way ANOVA demonstrated that there was a statistically significant difference in age across timepoints, $F(2, 1462) = 44.80$, $p < .001$, $\eta^2 = 0.058$. Post-hoc pair-wise comparisons revealed that participants were older in sample 1, as compared to sample 2 (0.40, (95% CI, 0.28 to 0.52), $p < .001$) and in sample 1 as compared to sample 3 (0.32, (95% CI, 0.27 to 0.41), $p < .001$). However, mean age did not significantly differ between samples 2 and 3 (-0.08, (95% CI, -0.20 to 0.04), $p = .26$).

Results of a chi-square test demonstrated that the distribution of male to female respondents differed significantly across timepoints, X^2 (2, N = 1419) = 25.871, $p < .001$,

Cramer's $V = .135$). Bonferroni-adjusted post hoc tests identified significant deviations from the null distribution at timepoints 1 and 3 (see table below).

Table S5.1. *Chi square analysis of gender distribution (male, female) at timepoints 1, 2 and 3*

			**Gender**		**Post hoc test results**
			Male	Female	X^2 (df, N)
**Timepoint**	1	Count	203	392	22.66 (1, 392)*
		Expected count	163.5	431.5	
		% within timepoint	34%	66%	
	2	Count	64	172	0.02 (1, 172)
		Expected count	64.9	171.1	
		% within timepoint	27%	73%	
	3	Count	123	465	21.72 (1, 465)*
		Expected count	161.6	426.4	
		% within timepoint	21%	79%	

Note. * denotes significant p-values < .001.

Acknowledgements:

Meg Attwood is an SWDTP ESRC-funded (1+3) PhD student. This research was supported by the Elizabeth Blackwell Institute, University of Bristol, and partly funded by the Wellcome Trust [Grant number - 204813/Z/16/Z]. For the purpose of Open Access, the author has applied a CC BY public copyright licence to any Author Accepted Manuscript version arising from this submission.

Correspondence to: Meg Attwood, School of Psychological Science, University of Bristol, 12A Priory Road, Bristol, BS8 1TU; +44 (0) 117 374 6633; meg.attwood@bristol.ac.uk

Data availability statement: Supplementary analyses and contextual information in support of our submission are provided in this online supplement. Due to the sensitivity of the data involved, these data are published as a controlled dataset at the University of Bristol Research Data Repository data.bris, at https://doi.org/10.5523/bris.2nit8ium1cuwy2merjyyoe2r47. The metadata record published openly by the repository at this location clearly states how data can be accessed by bona fide researchers. Requests for access will be considered by the University of Bristol Data Access Committee, who will assess the motives of potential data re-users before deciding to grant access to the data. No authentic request for access will be refused and re-users will not be charged for any part of this process.
